# Hydrogen–Deuterium Exchange Mass Spectrometry Identifies Local and Long-Distance Interactions within the Multicomponent Radical SAM Enzyme, PqqE

**DOI:** 10.1021/acscentsci.3c01023

**Published:** 2024-01-17

**Authors:** Wen Zhu, Anthony T. Iavarone, Judith P. Klinman

**Affiliations:** † Department of Chemistry and Biochemistry, Florida State University, Tallahassee, Florida 32306, United States; ‡ California Institute for Quantitative Biosciences, University of California, Berkeley, California 94720, United States; § Department of Chemistry, University of California, Berkeley, California 94720, United States; ∥ Department of Molecular and Cell Biology, University of California, Berkeley, California 94720, United States

## Abstract

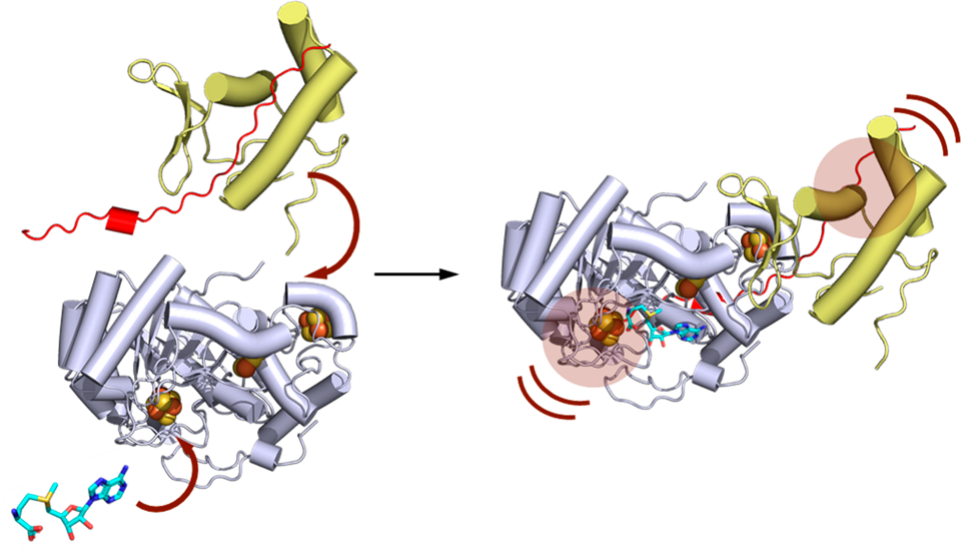

Interactions among proteins and peptides are essential for many biological activities including the tailoring of peptide substrates to produce natural products. The first step in the production of the bacterial redox cofactor pyrroloquinoline quinone (PQQ) from its peptide precursor is catalyzed by a radical SAM (rSAM) enzyme, PqqE. We describe the use of hydrogen–deuterium exchange mass spectrometry (HDX-MS) to characterize the structure and conformational dynamics in the protein–protein and protein–peptide complexes necessary for PqqE function. HDX-MS-identified hotspots can be discerned in binary and ternary complex structures composed of the peptide PqqA, the peptide-binding chaperone PqqD, and PqqE. Structural conclusions are supported by size-exclusion chromatography coupled to small-angle X-ray scattering (SEC-SAXS). HDX-MS further identifies reciprocal changes upon the binding of substrate peptide and S-adenosylmethionine (SAM) to the PqqE/PqqD complex: long-range conformational alterations have been detected upon the formation of a quaternary complex composed of PqqA/PqqD/PqqE and SAM, spanning nearly 40 Å, from the PqqA binding site in PqqD to the PqqE active site Fe_4_S_4_. Interactions among the various regions are concluded to arise from both direct contact and distal communication. The described experimental approach can be readily applied to the investigation of protein conformational communication among a large family of peptide-modifying rSAM enzymes.

## Abstract 2

HDX-MS detected local protection and remote conformational impact in the multicomponent radical SAM enzyme, PqqE, highlighting the importance of long-distance communication between the SAM and peptide binding sites.

## Introduction

Protein–protein and protein–peptide interactions are critical to many biological processes, such as receptor recognition and post-translational modification.^1,2^ Enzymes involved in the biosynthesis of ribosomally synthesized post-translationally modified peptides (RiPPs) possess an impressive capacity for installing functionally diverse modifications on their substrates.^3^ SPASM/twitch-domain-containing radical S-adenosylmethionine (rSAM) enzymes are an emerging class of RiPP-producing enzymes that use highly reactive radicals, generated by the reductive cleavage of S-adenosyl l-methionine (SAM), to activate C–H bonds in peptides.^4,5^ These rSAM enzymes catalyze a range of modifications, such as C–C, C–O, and C–S bond cross-links, epimerization, and oxidative decarboxylation.^6−14^ Harnessing the biochemical capabilities of rSAM enzymes has, in part, been hindered by a lack of knowledge of the specific interactions between a peptide substrate and its cognate rSAM enzyme. A thorough understanding of such interactions is expected to provide important insights into substrate utilization by rSAM enzymes and their potential application to biocatalysis.

**Figure 1:**
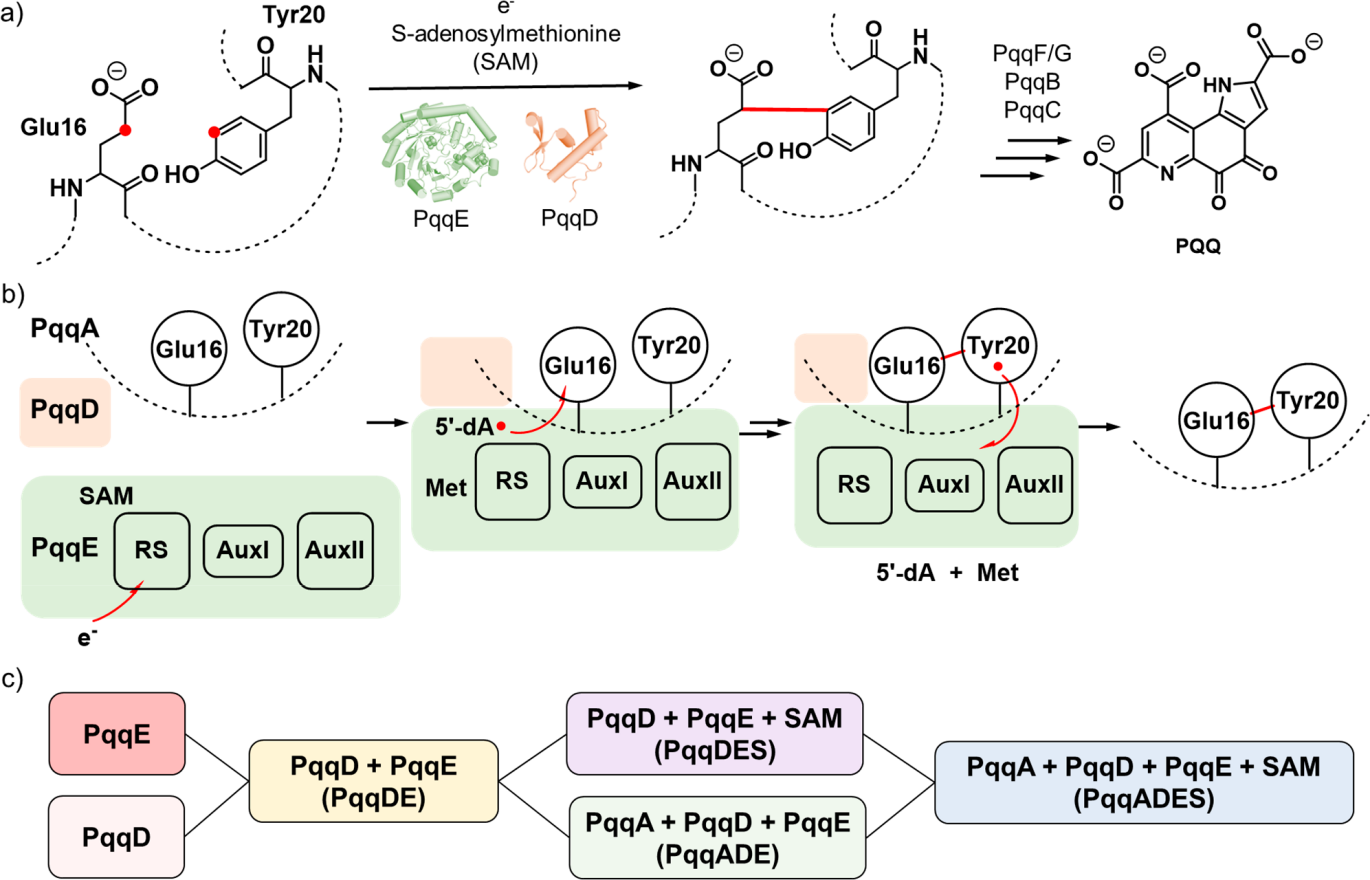
PQQ biosynthesis and reaction catalyzed by PqqE. a) Overview of PQQ biosynthesis. b) Reaction catalyzed by PqqE (green box) in which PqqA is first modified via a cross-linking reaction. The reduced RS Fe_4_S_4_ cluster of PqqE initiates reductive SAM cleavage to generate a 5′-dA radical that abstracts a hydrogen from Glu16 of PqqA that is bound to its chaperone PqqD (light-orange box). This leads to a C–C cross-linked product with Tyr20 (numbering refers to *Methylorubrum extorquens*). The AuxI and AuxII iron–sulfur clusters thought to shuttle electrons within PqqE are shown as black boxes adjacent to the RS site. c) Protein sample sets prepared and analyzed in this work. The designated name for each sample is shown in parentheses.

PqqE is one of the well-characterized RiPP-producing rSAM enzymes that contributes to the initial step of the biosynthesis of pyrroloquinoline quinone (PQQ).^6,15,16^ PQQ is a bacterial redox cofactor that is essential for several prokaryotic enzymes to metabolize C1 substrates. It has also been shown to promote the growth of bacteria and plants and to have beneficial impacts on the longevity of mammals.^17−20^ On the other hand, the *pqq* operon exists in many opportunistic bacterial pathogens, such as *Pseudomonas aeruginosa* and *Klebsiella pneumoniae*, but not in beneficial bacteria, including *Lactobacillus* and *Bifidobacterium*.^17^ Explicitly targeting the PQQ biosynthesis pathway by antimicrobial agents could therefore disarm opportunistic bacterial pathogens without damage to the healthy microbiome.^16^ Decades of effort have revealed that the biosynthetic pathway of PQQ (Figure 1a) begins with installation of a C–C cross-link between the side chain of a Glu and a Tyr on the precursor peptide, PqqA, by PqqE, in the presence of chaperone protein PqqD.^21^ Cross-linked PqqA is then processed by a protease (PqqF/G),^22^ an Fe-dependent hydroxylase (PqqB),^23^ and a cofactor-independent oxidase (PqqC)^24^ to produce PQQ.

PqqE belongs to the SPASM/twitch domain-containing subfamily of rSAM enzymes.^21^ The N-terminal TIM barrel domain of PqqE has a classical CxxxCxxC motif for the coordination of a Fe_4_S_4_ cluster, named the RS cluster (Figure 1b). This is the site where 5′-deoxyadenosyl radical (5′-dA·) is generated by the reductive cleavage of SAM when the RS cluster of PqqE is reduced by biological reductants such as flavodoxin/ferredoxin and NADP-flavodoxin/ferredoxin reductase.^21,25^ At the C-terminus, PqqE contains a SPASM domain that coordinates two auxiliary iron–sulfur clusters (AuxI and AuxII), which are essential for peptide modification activity^25^ (Figure 1b). Although these auxiliary iron–sulfur clusters are not required for the production of the 5′dA radical, their removal abolishes peptide cross-linking activity and severely impairs SAM cleavage at the RS site.^25^ We have used electron paramagnetic resonance (EPR) spectroscopy and electrochemistry to establish the redox properties of all iron–sulfur clusters in PqqE, leading to a working reaction mechanism that requires electron shuttling among iron–sulfur clusters^25,26^ (Figure 1b). Besides enabling the necessary “electronic communication”, our mutagenesis studies have suggested that iron–sulfur clusters may also contribute to the structural integrity of the SPASM domain, which could be important for protein–protein interactions in the PqqE-catalyzed reaction.^25^

Chemical cross-linking of PqqA requires that the precursor peptide, PqqA, binds to a chaperone protein, PqqD, to form a 1:1:1 ternary complex with PqqE in solution.^27^ In *Methylorubrum extorquens* AM1, PqqA is a short peptide containing 29 amino acids. As expressed from the operon, PqqD is a fusion protein attached to the C-terminus of PqqC. PqqC does not contribute to the first step of PQQ biosynthesis,^24^ and studies within have utilized a free PqqD construct. The affinity between PqqA and PqqD is relatively high (K_d_ = 0.39 ± 0.08 μM), while the interaction between PqqD and PqqE has been shown to be much weaker (K_d_ = 4.5 ± 1.5 μM).^27^ Although PqqD residues involved in the binding of PqqA and PqqE have been identified using solution nuclear magnetic resonance (NMR) measurements,^28,29^ the residues in PqqE responsible for the binding of PqqD and PqqA remain unknown. More importantly, it is unclear how the interactions among PqqA, PqqD, and PqqE lead to catalysis.

To address this question, we have investigated a combinatorial set of complexes involving SAM, PqqA, PqqD, and PqqE using time-dependent hydrogen–deuterium exchange mass spectrometry (HDX-MS) complemented by small-angle X-ray scattering (SAXS) analysis (Supporting Information Figure 1). HDX-MS has been widely used for mapping spatial interactions and dynamics in protein–protein and protein–peptide complexes.^30−32^ The altered deuterium uptake pattern probes the accessibility of backbone amides to solvent D_2_O; this can arise via direct protection at the interface of a protein complex or from alterations in conformational states due to distal binding interactions. We have applied this methodology to a series of binary, ternary, and quaternary complexes that are expected to form during the first step of PQQ biosynthesis; these represent complexes of PqqD/PqqE (PqqDE), PqqA/PqqD/PqqE (PqqADE), PqqD/PqqE/SAM (PqqDES), and PqqA/PqqD/PqqE/SAM (PqqADES) (Figure 1c). By comparing differences in HDX among the various components, we mapped the regions in PqqD and PqqE responsible for the interactions of each constituent in the PqqADES quaternary complex. Our results have led to a structural model for the ternary complexes and the detection of remote conformational changes in the formation of the quaternary complex. In comparison with the resolved crystal structures of other RiPP-rSAM enzymes, the presented solution-based structural model for PqqD suggests that analogous conformational rearrangements may be widely distributed within this subfamily of enzymes.

## Results and Discussion

### Peptides Derived from PqqE and PqqD Exhibit Time-Dependent Deuterium Uptake in the Absence of Binding Partners

Using PqqE or PqqD alone as the frame of reference, we first performed the time-dependent HDX-MS experiments at five time points (t_0_ = 0 min, t_1_ = 2 min, t_2_ = 9 min, t_3_ = 50 min, and t_4_ = 180 min). The sequence coverage for PqqE-derived peptides was 82.4% (Supporting Information Figure 2a). Mapping these peptides onto the PqqE structure (Supporting Information Figure 2b) shows that at the early time point, peptides derived from the TIM barrel region of protein exhibit less HDX in comparison to peptides derived from the SPASM domain, indicating differences in solvent accessibility within the SPASM domain and the active site residing within the TIM barrel of PqqE. We also observe a clear time dependency of deuterium uptake with labeling time for the TIM barrel-derived peptides. In samples containing only PqqD, the sequence coverage for PqqD-derived peptides was 90.4% under the same experimental conditions (Supporting Information Figure 2c). Increased deuterium incorporation in a time-dependent manner was similarly seen for peptides derived from PqqD after mapping them onto the solution NMR-derived structure of PqqD (Supporting Information Figure 2d). In free PqqD, folded regions exhibit lower relative HDX than loop regions, although, in general, the absolute % exchange for PqqD-derived peptides is higher than that for PqqE-derived peptides. Overall, we observe excellent peptide coverage and a clear trend of time dependence for HDX when PqqE and PqqD are studied in isolation.

### General Methodology for HDX Analysis of PqqE and PqqD in Binary, Ternary, and Quaternary Complexes

We next analyzed interactions among PqqA, PqqD, PqqE, and SAM by comparing the percentage of deuterium uptake when PqqE and PqqD are complexed with different partners. While catalysis is shown to proceed in the presence of a quaternary complex (PqqADES) following the addition of reductant, an analysis of precursor complexes, including PqqDE, PqqADE, and PqqDES, provides insight into the structure of the catalytically relevant complex prior to the initiation of reaction. HDX differences between any two complexes, defined as Δ%D, are calculated using the set of data at the same time points, where the subscript of Δ%D indicates peptides used for comparison and the superscript of Δ%D indicates the origin of the peptide from PqqE (e) or PqqD (d). A negative Δ%D value means that the additional component protects the peptide from HDX. Conversely, a positive Δ%D value shows that amides in the peptide are more prone to exchange in the presence of the additional component. Mapping these peptides onto the structures of PqqE and PqqD allowed us to locate the protein–protein interface by pairwise comparison as well as conformational rearrangement hotspots in the presence of additional components. We use volcano plots to present statistically significant^33^ changes between data sets, in which only differences exhibiting a *p* < 0.01 are highlighted.^34^

HDX-MS was conducted under standardized conditions (Materials and Methods, Supporting Information) with all peptides exhibiting EX2 exchange, that reflects rapid equilibration of proteins between open and closed states prior to a rate-limiting chemical replacement of H by D within the protein backbone.^35^ In order to analyze readily each of the protein component within the analyzed complexes, we maintained high concentrations, in the μM range, and at close to equimolar concentrations. Rapid equilibration between free species and their complexes is expected at the earliest time point, where based on the magnitude of previously measured K_d_ values^27^ both bound and unbound components are present and contribute to the net HDX. (See Notes in Supporting Information for concentrations of free and bound proteins under each condition.) The peptides with a statistically significant change, Δ%D (*p* < 0.01), were categorized by their time dependency. The type I exchange represents the first 2 min of incubation and will be the most sensitive to regions of protection arising from complex formation. The type II pattern (at 9, 50, and 180 min) refers to changes at later time points that are absent at t_1_. In consideration of type II patterns, several events may contribute to observed HDX progression. First, there is the possibility of slow incorporation into the protected regions of the complexes that reduces the Δ%D at longer times. Concomitantly, the dissociation of components from their respective complexes will lead to D incorporation within previously protected regions, resulting in a regression of the HDX pattern toward the unbound components. Of particular interest is that longer times may produce alternate positions for complex formation and/or conformational isomerizations that increase or decrease the pattern of HDX exchange relative to the free reference proteins. The same peptide, when analyzed from different complexes, can exhibit either type I or type II behavior. The classification for each peptide applies only to the experimental condition described within each section.

### PqqDE Binary Complex Adopts a “Side-On” Binding Mode

#### Impact of PqqD on PqqE in PqqDE (PqqDE-PqqE)

**Figure 2:**
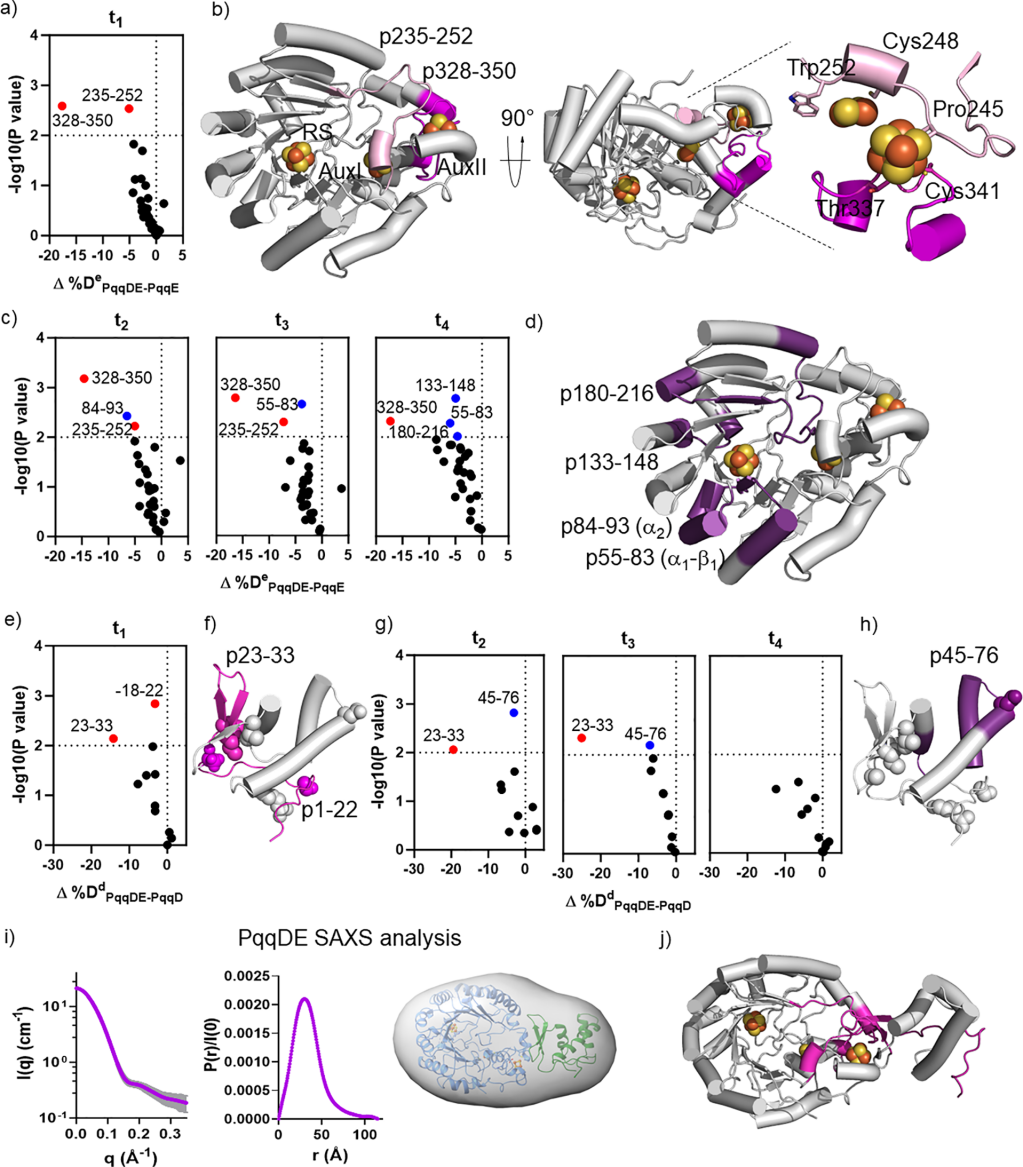
HDX of binary complex PqqDE. a) Volcano plot of Δ%D^e^_DE-E_ for PqqE-derived peptides at t_1_ obtained by comparing the %D HDX value for the complex PqqDE to that for PqqE alone (PqqDE-PqqE). Red dots represent type I peptides with *p* < 0.01. b) Two type I peptides, ^e^p235–252 (light pink) and ^e^p328–350 (magenta) are in the SPASM domain of PqqE. Missing loops and the RS Fe_4_S_4_ cluster in the X-ray structure for PqqE^39^ were modeled using AlphaFold^43,44^ and the CteB crystal structure.^7^ Iron–sulfur clusters are shown as spheres. c) Volcano plots of the Δ%D^e^_DE-E_ for PqqE-derived peptides at t_2_, t_3_, and t_4_. Red dots represent type I peptides with *p* < 0.01. Blue dots represent type II peptides with *p* < 0.01. d) Type II peptides, highlighted in purple, are located in the TIM barrel of PqqE. e) Volcano plot of Δ%D^d^_DE-D_ for PqqD-derived peptides at t_1_ obtained by comparing %D of HDX for the complex PqqDE to the value for PqqD alone (PqqDE-PqqD). Red dots represent type I peptides with *p* < 0.01. f) Two type I peptides, ^d^p(−18)–22 and ^d^p23–33 (magenta), are located in the N-terminal loop region and the first two β-sheets. PqqE binding residues identified by NMR are shown as spheres. g) Volcano plots of the Δ%D^d^_DE-D_ for PqqD-derived peptides at t_2_, t_3_, and t_4_. Red dots represent type I peptides with *p* < 0.01. Blue dots represent type II peptides with *p* < 0.01. h) Type II peptides cover an α-helical region in PqqD, including Asp-71, a previously proposed PqqE-binding residue. Type II peptide, ^d^p45–76, is highlighted in purple. PqqE binding residues identified by NMR are shown as spheres. i) SAXS data (left) and pair distance distribution analysis (middle) for the PqqDE complex. (Right) The electron density generated by DENSS^45^ (gray surface) matches the PqqD (green) and PqqE (light blue) complex in “side-on” mode. j) A PqqDE structural model constructed from HDX-MS analysis. The interface between PqqD and PqqE, as deduced from HDX, is shown in magenta.

To evaluate the impact of adding PqqD to PqqE, we compared the deuterium uptake of PqqE alone to PqqE in the PqqDE complex at each time point. Volcano plots identified ^e^p235–252 and ^e^p328–350 at the earliest time (type I exchange pattern), exhibiting −5% and −17% Δ%D^e^_DE-E_, respectively (Figure 2a). Mapping ^e^p235–252 and ^e^p328–350 onto the PqqE structure identifies a region of the SPASM domain that is strongly protected by PqqD (Figure 2b). Each of these two peptides contains one of the cysteine ligands of the iron–sulfur cluster of AuxI (Cys-248) and AuxII (Cys-341) as well as several conserved residues near AuxI, such as Trp-252, and AuxII, such as Pro-245, that do not appear in other RiPP-rSAM enzymes (Supporting Information Figure 3). These two peptides are connected to each other via a hydrogen bond between Ser-342 and Lys-243. The strong protection seen in these two peptides suggests a close interaction between the auxiliary iron–sulfur clusters and PqqD in their complex, perhaps providing a rationale for the complete loss of peptide modification activity when either AuxI or AuxII is knocked out by mutation.^25^ The type I peptides largely persist to varying degrees out to longer times. Four type II peptides, ^e^p55–83, ^e^p84–93, ^e^p133–148, and ^e^p180–216, are further identified in the volcano plots for HDX labeling times of t_2_ to t_4_ (Figure 2c). Peptides ^e^p55–83 and ^e^p84–93 are derived from α_1_-β_2_-α_2_ of the TIM barrel (Figure 2d). Although peptides ^e^p133–148 and ^e^p180–216 are also derived from the TIM barrel domain of PqqE, they exhibit significant changes only after 3 h of labeling.

#### Impact of PqqE on PqqD in PqqDE (PqqDE-PqqD)

Similarly, we determined the impact of PqqE on PqqD-derived peptides at each time point. PqqD-derived peptides, ^d^p(−18)–22 (N-terminal His-tag residues are numbered −18 to 0) and ^d^p23–33, exhibit significantly less deuterium uptake upon the addition of PqqE at t_1_, classified as type I (Figure 2e). Structurally, ^d^p23–33 represents a region composed of β-turns while ^d^p(−18)–22 is mostly from a flexible loop (Figure 2f). The Δ%D^d^_DE-D_ of ^d^p23–33 remains significant at later time points, while the Δ%D^d^_DE-D_ of ^d^p(−18)–22 becomes insignificant, despite the presence of PqqE. These observations are consistent with solution NMR measurements, which show that several residues in the N-terminal region of PqqD located in β-turns in PqqD respond to PqqE in the PqqADE complex^29^ (Figure 2f). The peptide ^d^p45–76 (Δ%D^d^_DE-D_ = −3%) appears as the only type II peptide in the comparison of PqqDE and PqqD at t_2_ (Figure 2g), showing an increased HDX difference at t_3_ (Δ%D^d^_DE-D_ = −7%) but becoming insignificant after 3 h. Mapping this peptide onto the PqqD structure identifies the segment connecting two α-helixes near the C-terminus of PqqD. Even though this peptide is not in close contact with the primary PqqE-interacting peptide ^d^p23–33, it includes Asp-71, a hotspot in the PqqD/PqqE interaction identified by NMR^29^ (Figure 2h). The insignificant Δ%D^d^_DE-D_ of ^d^p45–76 at longer labeling times in the HDX analysis is consistent with the small chemical shift perturbation of Asp-71 caused by PqqE in the NMR study, suggesting that this interaction is weak.

#### SEC-SAXS Supports the Side-On Binding Mode

As HDX protections are seen for the PqqE-derived peptides, ^e^p328–350 and ^e^p235–252, and the PqqD-derived peptides, ^d^p(−18)–22 and ^d^p23–33, at the same time points, it is likely that these peptides are located at the contact interface of the PqqDE complex. In this structural model, PqqE uses the SPASM domain to dock PqqD and PqqD interacts with PqqE via its N-terminal β-turns. To test this hypothesis, we performed size-exclusion chromatography coupled to small-angle X-ray scattering (SEC-SAXS) on the PqqDE complex (Figure 2i and Supporting Information Figure 4). The pair distance distribution plot of PqqDE indicates an elongated structure, consistent with a small subunit attached to a larger globular protein (Figure 2i), supporting a “side-on” binding model obtained from HDX analysis (Figure 2j).

#### Type II Peptides Suggest an Alternative Docking Pose for PqqDE

The HDX protection of the type II peptides clearly differs from the interaction between ^e^p235–252/^e^p328–350 and ^d^p23–33. Given the transient HDX changes in longer labeling time points for peptides from both components, ^d^p45–76 and ^e^p55–83/^e^p84–93, it is suggested that PqqD may also dock on PqqE at the α_1_-β_2_-α_2_ domain of the TIM barrel in a minor conformation.

### PqqADE Ternary Complex Identifies the PqqA Binding Regions on PqqE and PqqD

#### Impact of PqqA on PqqE in PqqADE (PqqADE-PqqDE)

**Figure 3:**
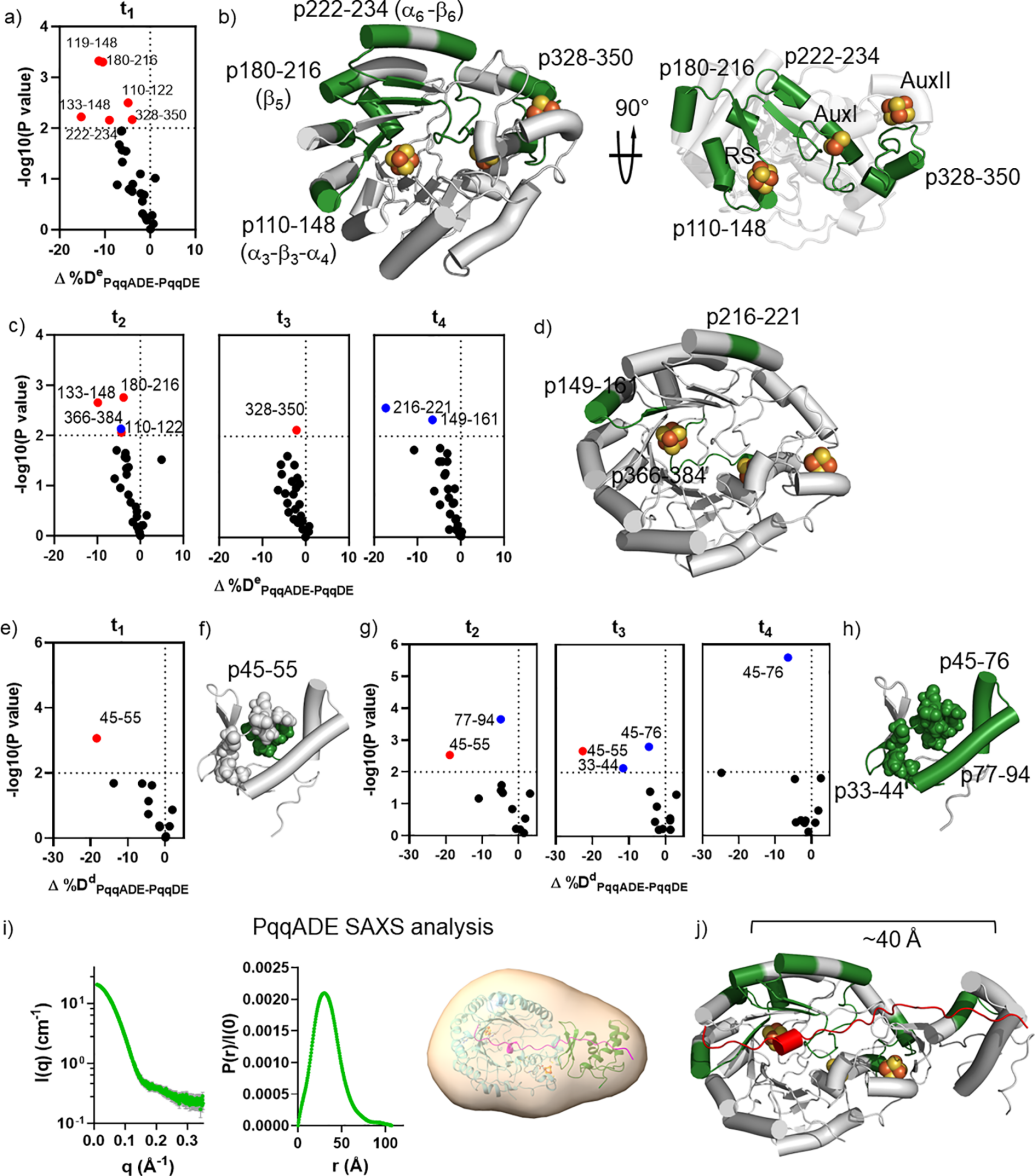
HDX of ternary complex PqqADE. a) Volcano plot of Δ%D^e^_ADE-DE_ values for PqqE-derived peptides at t_1_, obtained by comparing %D of HDX for the PqqADE complex to that of PqqDE (PqqADE-PqqDE). Red dots represent type I peptides with *p* < 0.01. b) Type I peptides (green) are located in the TIM barrel of PqqE. c) Volcano plots of the Δ%D^e^_ADE-DE_ for PqqE-derived peptides at t_2_, t_3_, and t_4_. Red dots represent type I peptides with *p* < 0.01. Blue dots represent type II peptides with *p* < 0.01. d) Mapping the PqqE-derived type II peptides (highlighted in green) on the structure. e) Volcano plot of Δ%D^d^_ADE-DE_ for PqqD-derived peptides at t_1_, obtained by comparing %D of HDX for the PqqADE complex to the value in PqqDE (PqqADE-PqqDE). f) The type I peptide (green), ^d^p45–55, is located in a helical region. PqqA binding residues identified by NMR are shown as spheres. g) Volcano plots of the Δ%D^d^_ADE-DE_ for PqqD-derived peptides at t_2_, t_3_, and t_4_. Blue dots represent type II peptides with *p* < 0.01. h) Type II peptides (green) contain all PqqA binding residues previously identified by NMR, shown as spheres. i) SAXS data (left) and pair distance distribution analysis (middle) for the PqqADE complex. (Right) Electron density generated by DENSS (orange surface) matches the PqqD (green), PqqE (light blue), and PqqA (magenta) complex in the side-on model. j) Proposed PqqADE structural model based on HDX analysis, in which the PqqA structure (red) has been predicted by AlphaFold to lack discrete secondary structure. The PqqA-protected region identified by HDX for both PqqD and PqqE is shown in green, and PqqA has been modeled on top of PqqE and PqqD accordingly.

The effect of adding PqqA on the deuterium uptake by PqqE in the PqqDE complex was investigated next. Many type I PqqE-derived peptides were identified that were protected by PqqA with statistically significant Δ%D^e^_ADE-DE_ values. These include ^e^p110–122 (−5%), ^e^p119–148 (−11%), ^e^p133–148 (−15%), ^e^p180–216 (−10%), ^e^p222–234 (−9%), and ^e^p328–350 (−4%). The Δ%D^e^_ADE-DE_ values at t_1_ decreased with increasing time for most of these type I peptides (Figure 3a). Mapping the type I peptides onto the PqqE structure reveals that both the TIM barrel and the SPASM domain are protected in the PqqADE complex when PqqA is present. Peptides exhibiting over 10% decreases in HDX are mostly located within β_3_-α and β_5_-α of the TIM barrel (Figure 3b). Most of the type I peptides disappear at later time points, as expected for transient protection between the PqqA and PqqE. Three PqqE-derived peptides, ^e^p149–161, ^e^p216–221, and ^e^p366–384, could be classified as type II (Figure 3c). Structural mapping shows that type II peptides are located mostly in the TIM barrel of PqqE (Figure 3d).

#### Impact of PqqA on PqqD in PqqADE (PqqADE-PqqDE)

Upon the addition of PqqA to the PqqDE complex, we identified PqqD-derived ^d^p45–55 (Δ%D^d^_ADE-DE_ = −18%) as a type I peptide (Figure 3e). This peptide is sandwiched between the N-terminal β-turns and C-terminal α-helixes in the NMR structure of PqqD (Figure 3f). Type II peptides from PqqD (Figure 3g) include ^d^p77–94 (Δ%D^d^_ADE-DE_ = −5%), ^d^p33–44 (Δ%D^d^_ADE-DE_ = −12%), and ^d^p45–76 (Δ%D^d^_ADE-DE_ = −6%). Importantly, these segments contain PqqA-perturbing residues previously assigned to be the PqqA binding region in the PqqAD complex by NMR (Figure 3h). Therefore, PqqA binds to the helical region of PqqD that represents a typical leader peptide-binding pocket seen in other peptide-modification enzymes.^36,37^

#### No Major Structural Changes for PqqDE upon PqqA Addition in SAXS

SAXS data were also obtained for the PqqADE complex (Figure 3i and Supporting Information Figure 4). The difference in SAXS between PqqDE and PqqADE, however, was small, suggesting that the side-on binding mode is maintained in the ternary complex. Using the established PqqDE side-on model and protection hotspots seen upon PqqA addition, we identified a connected region across PqqDE that spans nearly 40 Å from PqqD to the RS Fe_4_S_4_ cluster in PqqE. Although there is no experimental structure for PqqA, we estimated the distance between Met-1 to Glu-16 of PqqA to be approximately 43 Å based on an AlphaFold^38^ prediction (Supporting Information Figure 5). This distance is consistent with the model of the PqqADE complex proposed here based on HDX and SAXS measurements (Figure 3j). Because Glu-16 and Tyr-20 are located near the C-terminus of PqqA, we believe that the protection seen for PqqD is associated with the N-terminal leader peptide of PqqA. Equally, the TIM barrel protection in PqqE likely arises from interactions with the C-terminal region of PqqA.

#### PqqA Interacts with Both the SPASM Domain and the TIM Barrel of PqqE

In the crystal structure of PqqE,^39^ residues between 127 and 140 are poorly resolved, suggesting that this region is mobile. The high degree of protection of ^e^p119–148 and ^e^p133–148 within 2 min after PqqA addition, however, shows that the flexibility of this region is altered in the presence of PqqA (Figure 3a). In the AlphaFold model of PqqE, ^e^p110–148 is predicted to contain an α-helix (Supporting Information Figure 6) that could hydrogen bond with the CxxxCxxC motif near the RS Fe_4_S_4_ cluster. Peptides ^e^p180–216 and ^e^p222–234 cover the β_5_-α_6_-β_6_ secondary structural element of the TIM barrel. The loop region in ^e^p180–216 contains a highly conserved Gln-194, Tyr-196, and Trp-198 (QxYxW) triad located near the AuxI Fe_2_S_2_ cluster (Figure 3b). Aligning representative SPASM-rSAM enzyme sequences confirms that the QxYxW triad is a unique feature in PqqE (Supporting Information Figure 3). Interestingly, AlphaFold predicts that ^e^p180–216 contains an α-helix, moving the QxYxW triad closer to the RS Fe_4_-S_4_ cluster (Supporting Information Figure 6a). This is consistent with our previous demonstration of the importance of electronic communication between the AuxI and the RS Fe_4_-S_4_ cluster in the catalytic mechanism of PqqE.^25^ Type I peptide ^e^p328–350 is a SPASM domain peptide assigned to a PqqD docking site. In addition to the 17% HDX protection caused by the protection of PqqD alone (PqqDE-PqqE), PqqA binding contributes an extra 4% protection of HDX at ^e^p328–350 in PqqADE-PqqDE (Figure 2a). This observation is consistent with surface plasmon resonance measurements showing that K_d_ between PqqE and PqqD decreases 3-fold in the presence of PqqA.^27^ Two plausible explanations can explain this analysis: 1) protection comes from the direct interaction between PqqA and PqqE or 2) the binding of PqqA tightens up the site of the PqqDE interaction near AuxII. We consider the first scenario to be more likely, given the insignificant HDX changes of the PqqD-derived peptide at the interface of the PqqDE complex on the same time scale. Overall, these HDX experiments uncover three connected PqqE regions that span the RS, AuxI, and AuxII sites that are impacted by PqqA in the PqqADE complex. Although the interaction is perhaps transient, the extent of coverage demonstrates a conformational interdependence among the three iron–sulfur clusters.

### HDX of PqqDES Ternary Complex Shows a Typical SAM Binding Region in PqqE

#### Impact of SAM on PqqE in PqqDES (PqqDES-PqqDE)

**Figure 4:**
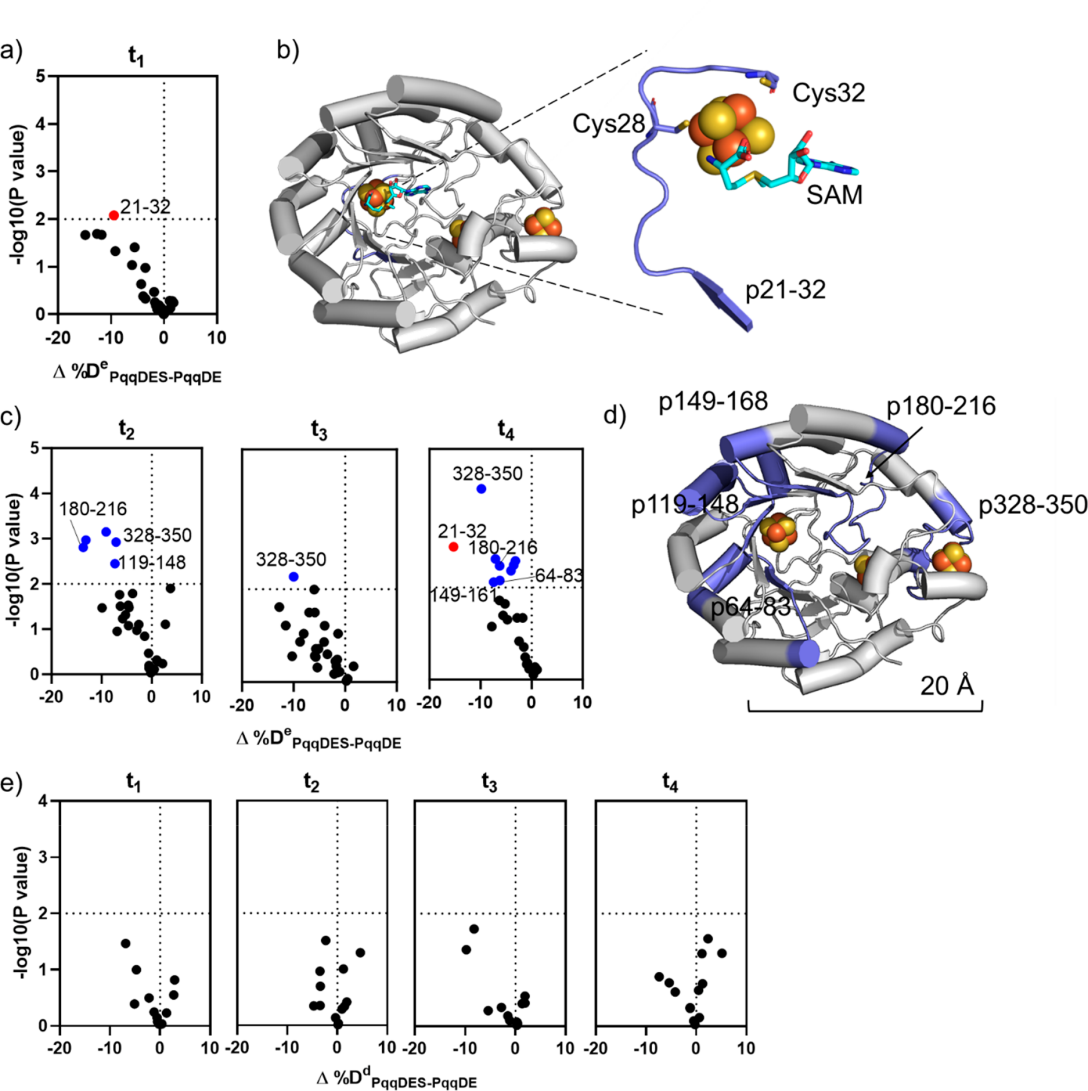
HDX of the PqqDES ternary complex. a) Volcano plot of Δ%D^e^_DES-DE_ for PqqE-derived peptides at t_1_, obtained by comparing %D of HDX for the complex PqqDES to the value of PqqDE. Red dot represents type I peptide with *p* < 0.01. b) Type I peptide (light purple) is located at the RS site of PqqE. The RS loop, the RS Fe_4_S_4_ cluster (orange/yellow spheres), which is missing in the crystal structure, and SAM (cyan) were modeled using AlphaFold and the CteB crystal structure. c) Volcano plots of the Δ%D^e^_DES-DE_ for PqqE-derived peptides at t_2_, t_3_, and t_4_. Red dot represents type I peptide with *p* < 0.01. Blue dots represent type II peptides with *p* < 0.01. d) Type II peptides (light purple) are mostly located in the TIM barrel of PqqE. Overlapping peptides are not labeled. e) Volcano plots of Δ%D^d^_DES-DE_ for PqqD-derived peptides at all time points, obtained by comparing %D of HDX for the complex PqqDES to the value of PqqDE.

When SAM was added to the PqqDE complex, we identified one type I peptide, ^e^p21–32 (Δ%D^e^_DES-DE_ = −9%), showing a reduced rate of HDX in the PqqDES complex (Figure 4a). This peptide contains two cysteines that are part of the signature CxxxCxxC motif of rSAM enzymes, Cys-28 and Cys-32, and which coordinate the RS Fe_4_S_4_ cluster (Figure 4b). The Δ%D^e^_DES-DE_ volcano plot showed that more peptides were impacted by the presence of SAM at t_2_ (Figure 4c). These include TIM-barrel-derived peptides, ^e^p64–83 (Δ%D^e^_DES-DE_ = −6%), ^e^p119–148 (Δ%D^e^_DES-DE_ = −7%), ^e^p149–161 (Δ%D^e^_DES-DE_ = −7%), and ^e^p180–216 (Δ%D^e^_DES-DE_ = −14%), and the SPASM domain-derived peptide, ^e^p328–350 (Δ%D^e^_DES-DE_ = −7%). These type II peptides show that the SAM-impacted region in PqqE extends beyond the SAM binding site (Figure 4d). Among these peptides, ^e^p119–148 contains Ser-123, which is highly conserved across all rSAM enzymes (Supporting Information Figure 3). The cognate serine interacts with the carboxylate and the ribo-hydroxide in SAM in other radical SAM enzymes, such as CteB.^7^ Peptide ^e^p64–83 contains the signature sequence “GGEP” located near the RS site, which is also highly conserved. Finally, ^e^p328–350, the peptide identified as involving PqqD binding in the absence of SAM, which is 20 Å from the RS Fe_4_S_4_ cluster, also shows increased HDX protection upon SAM addition. These observations strongly suggest that there is cooperativity between SAM binding in the TIM barrel and the SPASM domain of PqqE, possibly mediated by the loop present in ^e^p180–216 (Figure 4d).

#### Impact of SAM on PqqD in PqqDES (PqqDES-PqqDE)

The addition of SAM to the PqqDE complex did not result in significant HDX changes to any PqqD-derived peptide regardless of the labeling time (Figure 4e). The presented HDX results of PqqDES-PqqDE rule out any major conformational change within PqqD at the PqqDE interface upon SAM binding in the ternary complex.

#### SAM Protects the RS Binding Site and Induces HDX Protection near the PqqDE Interface and Does Not Alter the Binding of PqqD

The increased HDX protection at ^e^p21–32 upon SAM addition can be explained by the SAM coordination at the RS Fe_4_S_4_ cluster (Figure 4b). The HDX changes of PqqE-derived peptide ^e^p328–350 at the PqqDE interface upon SAM binding (Figure 4d), however, were not accompanied by any significant change in HDX of a PqqD-derived peptide. This suggests that the conformational rearrangement is localized within PqqE when the peptide substrate is absent. Overall, the impact of SAM on the HDX of PqqE-derived peptides in the PqqDES complex is consistent with crystal structures of other rSAM enzymes, such as SuiB^40^ and CteB,^7^ where SAM is exclusively bound to the RS Fe_4_S_4_ cluster and does not significantly impact the leader peptide binding domain. The broader impact of SAM addition is also limited to only PqqE and does not appear to impact the interaction between PqqE and PqqD.

### SAM Addition in the Presence of PqqA Alters Interactions among Components of the Quaternary Complex PqqADES

#### Impact of SAM on PqqE in PqqADES (PqqADES-PqqADE)

**Figure 5:**
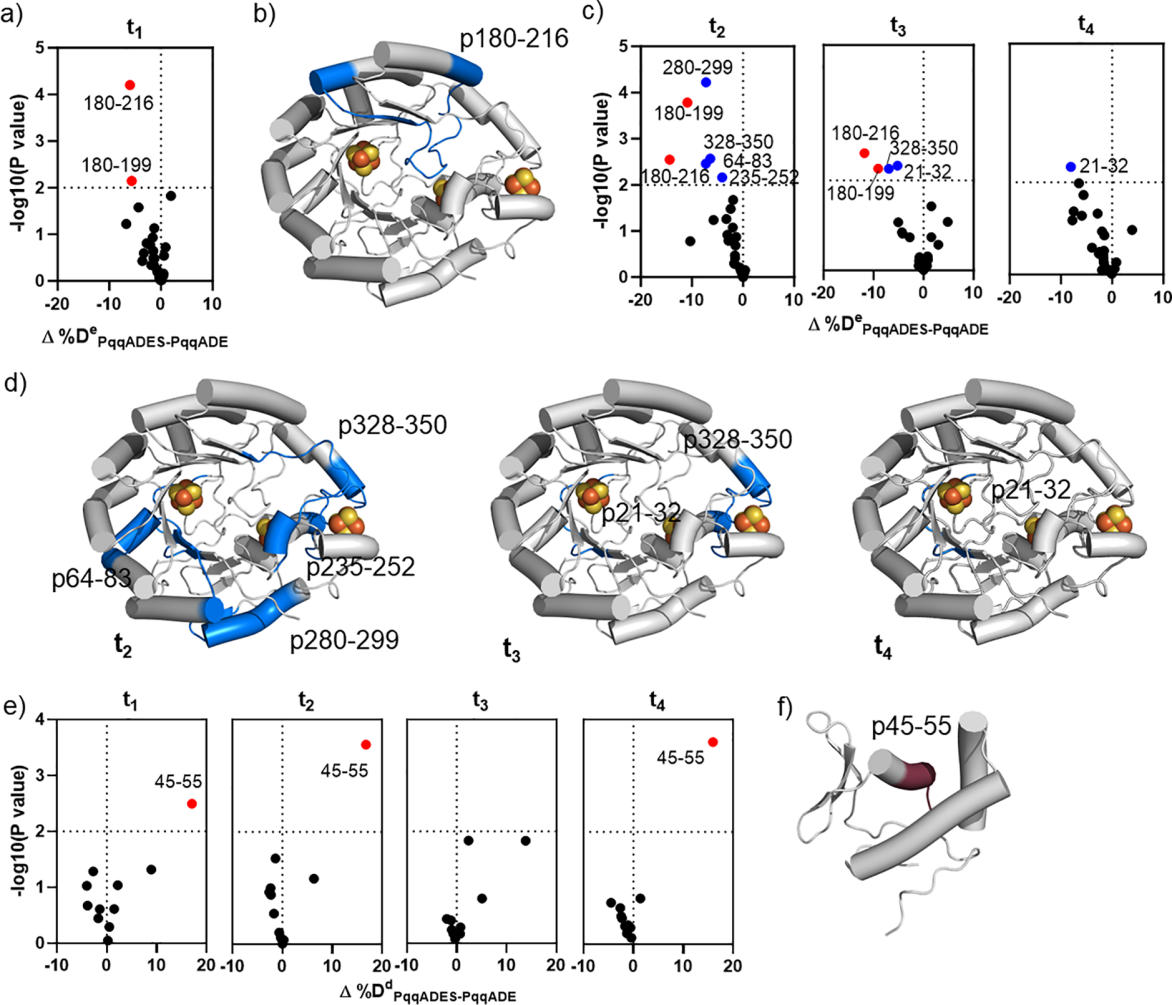
HDX of quaternary complex PqqADES in comparison with PqqADE. a) Volcano plot of Δ%D^e^_ADES-ADE_ for PqqE-derived peptides at t_1_, obtained by comparing %D of HDX for the complex PqqADES to the value of PqqADE. Red dots represent type I peptides with *p* < 0.01. b) Type I peptide (blue), ^e^p180–216, mapped on the structure of PqqE. c) Volcano plots of Δ%D^e^_ADES-ADE_ for PqqE-derived peptides at t_2_, t_3_, and t_4_. Blue dots represent type II peptides with *p* < 0.01. d) Type II peptides (blue) are mostly located in the TIM barrel of PqqE. The time dependence of the type II exchange pattern is highlighted. e) Volcano plots of Δ%D^d^_ADES-ADE_ for PqqD-derived peptides at all time points, obtained by comparing %D of HDX for the complex PqqADES to the value of PqqADE. f) Mapping ^d^p45–55 (raspberry) onto the PqqD NMR structure is consistent with the assigned PqqA binding pocket (Figure 3f); this peptide becomes more solvent-exposed on SAM binding.

We next evaluated how SAM impacts PqqE in the presence of PqqA and PqqD. Type I peptides, identified in the comparison of deuterium uptake in the PqqADES and PqqADE complexes, include overlapping peptides ^e^p180–199 (Δ%D^e^_ADES-ADE_ = −6%) and ^e^p180–216 (Δ%D^e^_ADES-ADE_ = −6%) (Figure 5a). Peptide ^e^p180–216 is derived from the region of the TIM barrel (Figure 5b) that is also protected by the addition of SAM in the absence of PqqA, although the time dependence of HDX identified by PqqADES-PqqADE analysis clearly differs from what is seen for PqqDES-PqqDE (Figure 4c). Five type II peptides, including Δ%D^e^_ADES-ADE_^e^p280–299 (−7%) that is distal from the iron-sulfur clusters, showed significant analyzable Δ%D^e^_ADES-ADE_ at t_2_ and longer time points when SAM was added to the PqqADE complex (Figure 5c). Peptides ^e^p235–252 and ^e^p328–350, which are implicated in the PqqD/PqqE interaction (Figure 2b), exhibited greater protection, −4 and −6% of Δ%D^e^_ADES-ADE_, respectively. Peptide ^e^p21–32 (Δ%D^e^_ADES-ADE_ = −8%) was identified in the sample only after t_3_, however, when most of the type II peptides are no longer seen in the volcano plots (Figure 5c). Fewer peptides exhibit a type II exchanging pattern as the labeling time increased. This trend may result from deuterium labeling during the dissociation of free from bound states. It can also suggest the flexible nature of the quaternary complex (Figure 5d). These trends clearly differ from the comparison between PqqDES and PqqDE (Figure 4d).

#### Impact of SAM on PqqD in PqqADES (PqqADES-PqqADE)

To examine how SAM influences the HDX of PqqD in the PqqADES quaternary complex, we compared HDX in PqqD-derived peptides from PqqADES and PqqADE (Figure 5e). *A particularly significant observation is that peptide^d^p45–55, the only identified type I peptide for PqqD, exhibits a positive Δ%D value (Δ%D^d^_ADES-ADE_= +17%).* Furthermore, ^d^p45–55 is located in the α-helix assigned as the PqqA binding site by NMR studies^29^ (Figure 5f).

#### SAM Alters Interactions with PqqA within the PqqADES Complex

In the presence of PqqA, we have seen a different HDX pattern at early times in relation to PqqDES-PqqDE (Figures 5a and 4a). First, the addition of SAM does not alter the Δ%D^e^_ADES-ADE_ of ^e^p21–32, the key region shown to be protected at t_1_ by SAM in the absence of PqqA. As discussed immediately above, at early times protection at the SAM binding pocket is altered in peptide ^e^p180–216 as well as in a distal region near the AuxII site (peptide ^e^p235–252). Another example that distinguishes PqqADES-PqqADE and PqqDES-PqqDE is that ^e^p280–299 is not associated with any binary or ternary complex and becomes protected only when both substrates are present. These data suggest that the binding of the second substrate alters the conformation of PqqE from what is present after binding the first substrate.

In the comparison of PqqD-derived peptides, ^d^p45–55 shows a positive Δ%D^d^ value, suggesting more exposure to D_2_O upon SAM addition to PqqADE (Figure 5e). Increased amide exchange in this region of PqqD at various labeling time points indicates that SAM binding at the RS Fe_4_S_4_ site of PqqE changes the HDX pattern of the peptides previously assigned as the PqqA binding pocket in PqqD. This can result from a weakened interaction between PqqA and PqqD. Alternatively, it could indicate long-distance communication between the SAM binding site in PqqE and the conformational properties of PqqD in the quaternary complex (Supporting Information Figure 5). No other peptides, such as ^d^p33–44 and ^d^p77–94, exhibit a loosening effect in the presence of SAM binding, suggesting that the remainder of the PqqA leader sequence maintains its interaction with PqqD.

### PqqA Changes the PqqE/SAM Interaction in the PqqADES Quaternary Complex

#### Impact of PqqA on PqqE in PqqADES (PqqADES-PqqDES)

**Figure 6:**
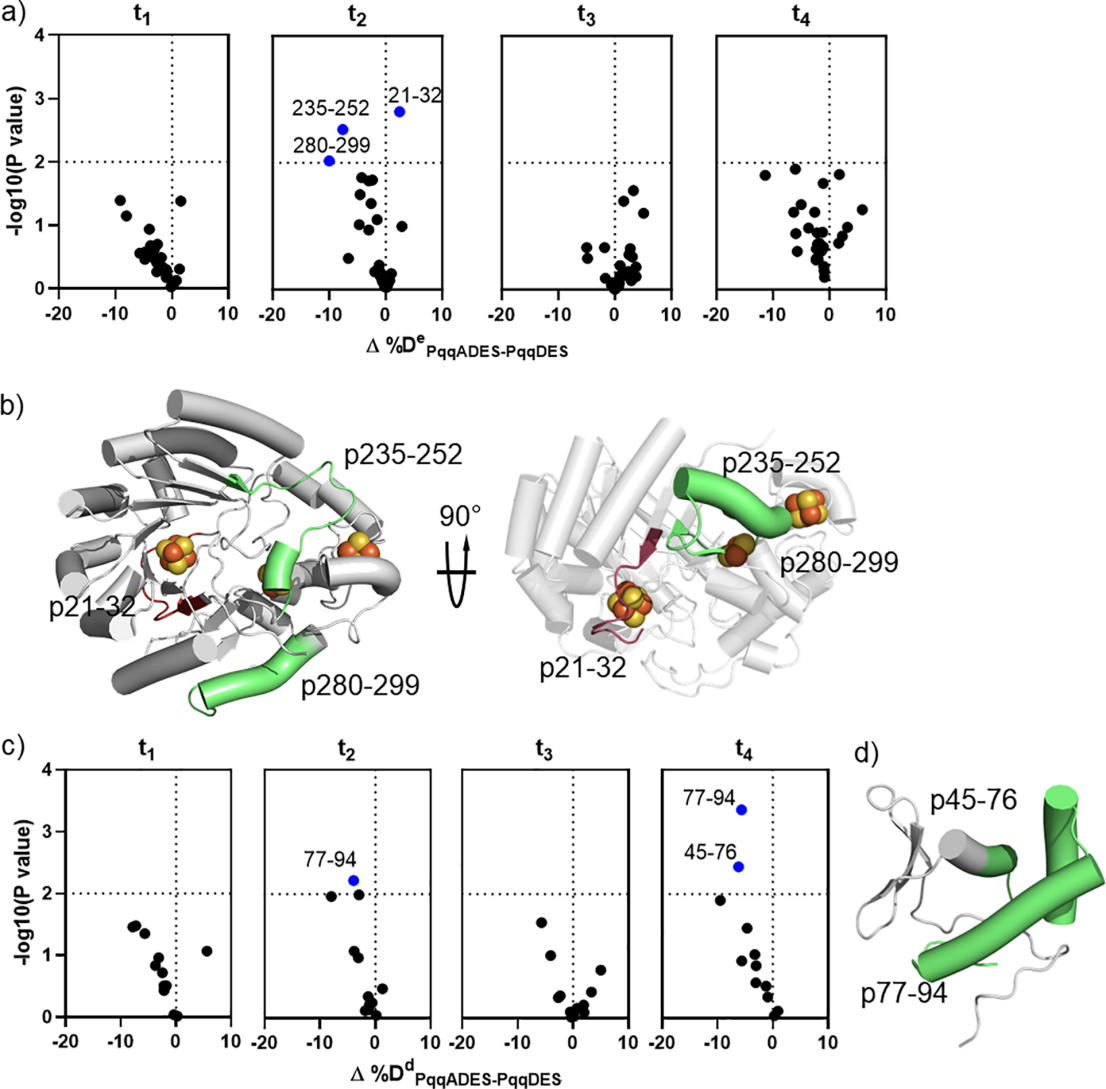
HDX of quaternary complex PqqADES in comparison with PqqDES. a) Volcano plots of Δ%D^e^_ADES-DES_ for PqqE-derived peptides at t_1_, obtained by comparing %D of HDX for the complex PqqADES to the value of PqqDES. Blue dots represent type II peptides with *p* < 0.01. b) Type II peptides with negative Δ%D^e^_ADES-DES_ (light green) mapped onto the structure of PqqE. The peptide, ^e^p21–32, exhibiting positive Δ%D^e^_ADES-DES_ is highlighted in raspberry. c) Volcano plots of Δ%D^d^_ADES-DES_ for PqqD-derived peptides at all time points obtained by comparing %D of HDX for the complex PqqADES to the value of PqqDES. Blue dots represent type II peptides with *p* < 0.01. d) Mapping type II peptides onto the NMR-derived structure (light green) shows the PqqA binding pocket in PqqD that becomes more protected upon PqqA addition.

No type I peptide shows a significant HDX change for PqqE when PqqA is added to the PqqDES complex (Figure 6a). Three type II peptides are identified at t_2_, for which HDX differences become insignificant at later time points (Figure 6a). Two of these peptides ^e^p235–252 and ^e^p280–299 show increased protection within the SPASM domain, −7 and −10% of Δ%D^e^_ADES-DES_, respectively. *In contrast, the Δ%D^e^_ADES-DES_for^e^p21–32 is +2%, indicating that SAM binding to PqqE is altered, leading to greater exchange in the presence of PqqA* (Figure 6b). As already noted, ^e^p21–32 is part of the RS Fe_4_S_4_ cluster binding motif, where the 5′dA radical is formed. This indicates that PqqA changes the solvent exposure at the SAM binding site.

#### Impact of PqqA on PqqD in PqqADES (PqqADES-PqqDES)

Two type II peptides, ^d^p77–94 (Δ%D^d^_ADES-DES_ = −4%) and ^d^p45–76 (Δ%D^d^_ADES-DES_ = −6%), are identified in the comparison between PqqADES and PqqDES (Figure 6c) and are assigned as being part of the PqqA binding region in PqqD (Figure 6d).

#### PqqA Reduces the SAM-Induced Protection in the RS Binding Region in PqqE in the PqqADES Complex While Showing Altered Interactions with PqqD

In contrast to the behavior of other complexes, no type I behavior is observed in this case. Perhaps the most interesting finding from comparing the PqqADES and PqqDES complexes is that ^e^p21–32 at t_2_ exhibits +2% of Δ%D^e^_ADES-DES_ (Figure 6a). This shows that the addition of PqqA can partially weaken the protection provided by SAM at ^e^p21–32 of PqqE in the complex containing both substrates. At longer time points, however, none of the Δ%D^e^_ADES-DES_ values are statistically significant, indicating that both greater exposure and protection are lost and strongly suggestive of weak interactions that can be detected only at earlier times.

Nonetheless, the type II peptides, ^e^p235–252 (in the region of PqqD binding (Figure 2b)) and ^e^p280–299 (distal from Fe–S centers (Figure 5d), which have been associated with PqqD and SAM binding, respectively, are impacted by binding PqqA (Figure 6b). This observation is consistent with the model in which the addition of PqqA to the PqqDES complex rearranges regions not only within PqqD but also within PqqE. For the PqqD-derived peptides, the α-helixes of PqqD have been assigned to be the PqqA binding site based on both type I peptides in the PqqADE-PqqDE analysis (Figure 3h). However, in PqqADES-PqqDES, only type II peptides are detected in the same region, and ^d^p45–55 shows no significant difference in HDX at t_1_ (Figure 6c). As it is highly unlikely that SAM directly binds to the PqqA-binding site in PqqD, it is likely that the interaction between PqqA and PqqD at ^d^p45–55 is weakened when SAM is present in PqqE. This is consistent with the hypothesis of a conformational rearrangement upon quaternary complex formation. Taken together, the analysis of PqqADES-PqqDES supports a conformational change upon formation of the quaternary complex PqqADES that includes a weakening of the interaction between SAM and the RS site at position ^e^p21–32 and the interaction between PqqA and PqqD at position ^d^p44–45.

### HDX-Derived Structural Model of PqqADES in Comparison with X-ray Crystal Structures of Related Enzymes

Crystal structures of substrate-enzyme complexes have been obtained for several other peptide modification SPASM/twitch-rSAM enzymes catalyzing the cyclization of peptides, such as SuiB,^40^ CteB,^7^ and SkfB.^41^ In these structures, SAM always binds to the RS site, although different binding modes have been observed.^7,40^ The leader peptide binding regions and the relative location of their RiPP recognition element (RRE, equivalent of PqqD) in rSAM, however, vary in these complexes. More specifically, CteB binds the leader peptide of its substrate via its RRE domain, while SuiB uses its SPASM domain to anchor its peptide substrate. Previously, it was challenging to simply transfer their protein–protein and protein–peptide interactions to the PqqE system due to the differences in peptide length and iron–sulfur clusters as well as the fact that the RRE in CteB, SuiB, and SkfB are all covalently attached to the rSAM enzyme. Our time-dependent HDX-MS analyses of PqqE and PqqD, in the presence or absence of SAM and PqqA, provide rich, spatially resolved information about changes in PqqD/PqqE interactions when these proteins form catalytically relevant complexes. First, our solution-based structural model confirmed that the PqqDE interaction is through the β-sheets in PqqD and the SPASM domain in PqqE, which is consistent with SuiB, CteB, and SkfB. The side-on model suggests that the PqqDE complex resembles the crystal structure of CteB (Supporting Information Figure 7a). Second, we have also identified that the CxxxCxxC motif near the RS site is sensitive to PqqA-induced conformational changes (Figure 6b). In SuiB, a loop near the RS site, L1 (SuiB p125–134), in the rSAM domain adopts alternative conformations upon quaternary complex formation^40^ (Supporting Information Figure 7b). Finally, we observe evidence for an alternative PqqD protection site near α_1_-β_2_-α_2_ of the TIM barrel in PqqE (Figure 2d). This region corresponds to the RRE-rSAM interface in the crystal structures of SuiB and SkfB (Supporting Information Figure 7c). Thus, our HDX-derived complex model suggests the coexistence of a minor conformation in solution, which is supported by crystal structures of SuiB and SkfB. In combination, both HDX and X-ray methods capture snapshots that implicate dynamically active protein complexes in the catalytic turnover of rSAM enzymes.

### Proposed Conformational Change upon Protein Complex Formation

The negative Δ%D values in the HDX-MS analyses of PqqE- and PqqD-derived peptides identify the interaction sites between binding partners within a range of binary, ternary, and quaternary complexes. The positive value of Δ%D^e^_ADES-DES_ for the PqqE-derived peptide ^e^p21–32 and the Δ%D^d^_ADES-ADE_ for the PqqD-derived peptide ^d^p45–55 both point to substrate-induced conformational changes upon the formation of the quaternary complex containing PqqA, PqqD, PqqE, and SAM. A major synergistic rearrangement is detected, spanning ca. 40 Å between the RS site in PqqE and the PqqA-binding pocket of PqqD. This rearrangement occurs only in the presence of both PqqA and SAM.

**Figure 7:**
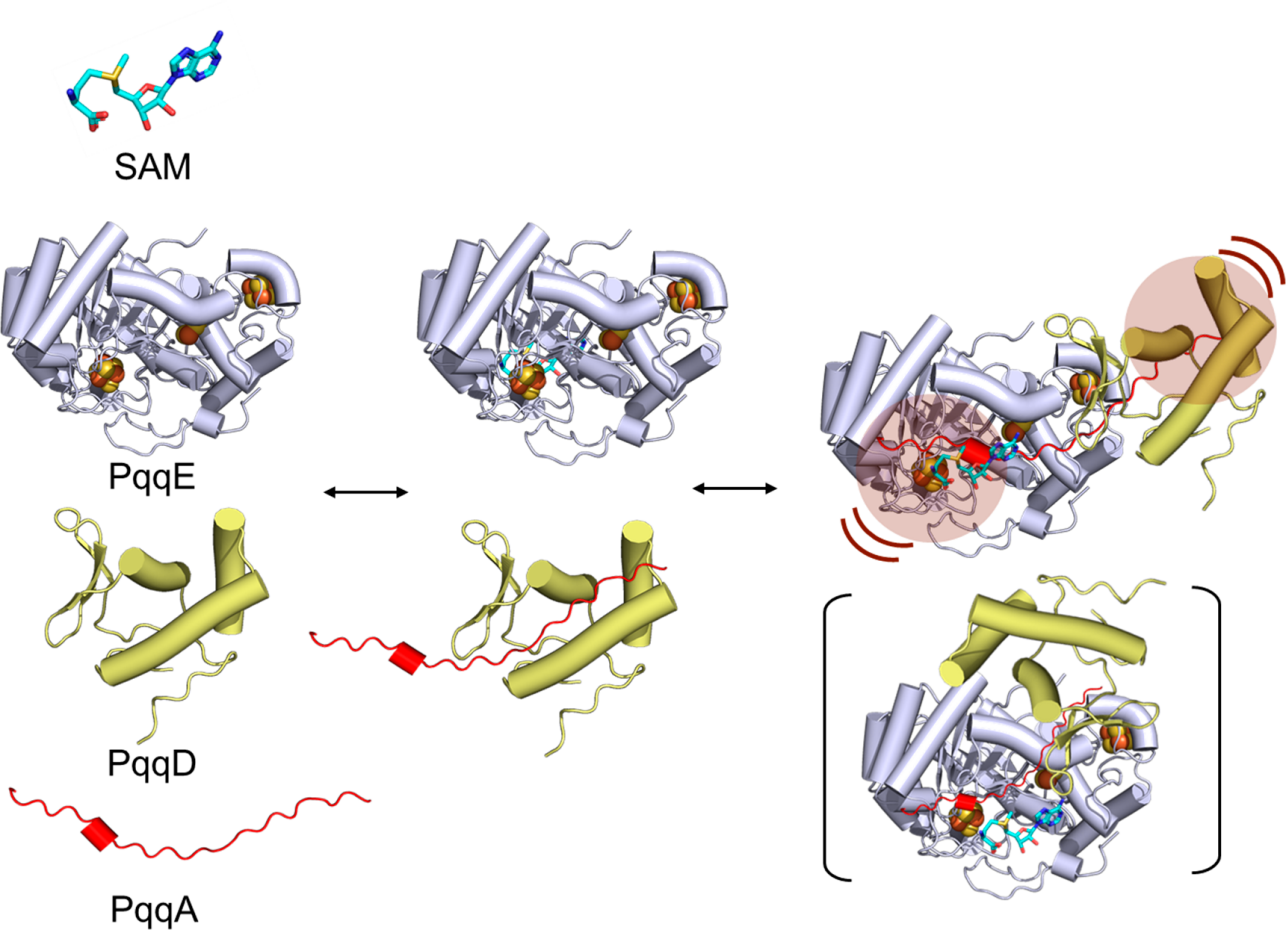
Proposed quaternary complex formation. Binding of SAM (cyan sticks) and PqqA (red) in PqqE (light purple) and PqqD (yellow) leads to reciprocal effects (highlighted in the raspberry shaded circle) on PqqD and PqqE, respectively. The model includes an extended conformation for the bound PqqA that stretches from its tethered site on PqqD toward the rSAM catalytic site. A second minor conformation detected for PqqDE is shown in parentheses, as this may play a role in the initiation of the substrate-dependent reductive cleavage of SAM within a quaternary complex.

In Figure 7, we present a conformational rearrangement model resulting from the binding of PqqA and SAM to PqqD and PqqE, respectively, to form the catalytically ready form of PqqADES. Adopting the previously proposed PqqAD interaction model,^29^ the leader sequence of PqqA interacts with the α-helical regions of PqqD. This PqqAD complex likely delivers the reactive Glu-16 and Tyr-20 to PqqE via the docking site at the SPASM domain of PqqE. The binding of SAM at the RS site of PqqE could be independent of the PqqDE and PqqAD interaction. Upon the formation of the quaternary PqqADES complex, the C-terminal region of PqqA alters the conformation of the RS site of PqqE. In a reciprocal fashion, SAM binding alters the interaction between PqqD and PqqA, allowing PqqD to loosen/release the leader peptide of PqqA while possibly opening up a new site for complexation of PqqD to PqqE in a position that resembles the substrate peptide binding site seen in the crystal structure of SuiB (Supporting Information Figure 4c).

From the present focus on the quaternary complex of PqqE with its accessory protein, peptide substrate, and SAM, it is possible to define a precursor ground-state structure for enzymatic activity. While this PqqADES quaternary complex itself is catalytically inactive, productive turnover of PqqE takes place upon the addition of a flavodoxin/ferredoxin with an NADP-dependent flavodoxin/ferredoxin reductase pair.^21,25^ However, the native reductant for the PQQ biosynthetic pathway remains unknown, and the use of dithionite as a chemical reductant leads to a complete uncoupling of SAM cleavage from peptide activation.^21,42^ Understanding the impact of different reducing reagents on the structure and conformational plasticity of rSAM enzymes remains an outstanding question and challenge. In this context, the established model (Figure 7) provides a robust basis for further interrogation of how reductants may alter protein complex structure and dynamics.

## Conclusions

We have applied time-dependent HDX-MS and SEC-SAXS to establish a quaternary structural model for the multicomponent peptide modifying rSAM enzyme in PQQ biosynthesis. Our model reveals long-distance cooperativity in the enzyme quaternary complex, spanning a distance of ∼40 Å. Induced substrate restructuring across the entire protein complex can be envisioned, which not only accommodates the catalytically relevant states but may also prevent potentially damaging side reactions during the challenging free radical chemistry. The striking range of interactions embedded in PqqE may serve as a template for understanding rSAM enzymes that function on peptide substrates. The presented results go a long way toward clarifying the multiple sites and conformational interactions that generate a catalytically functional PqqADES complex.

## Supporting Information Available

Materials and methods, supplementary notes, supplementary Figures 1 to 7, and SI references (PDF)
Data set: HDX data summary table (XLSX)
Data set: Time-dependence of each peptide reported in this work (PDF)
