# Supplementary material for: Hydrogen–Deuterium Exchange Mass Spectrometry Identifies Local and Long-Distance Interactions within the Multicomponent Radical SAM Enzyme, PqqE
Source: ACS Cent Sci. 2024 Jan 17;10(2):251–63. doi: 10.1021/acscentsci.3c01023 (PMC10906245; doi:10.1021/acscentsci.3c01023)
Supplement: Supplementary file 1 — oc3c01023_si_001.pdf [file oc3c01023_si_001.pdf]

Supplementary Information

Hydrogen-deuterium exchange mass spectrometry identifies local and long-distance interactions within the multi-component radical SAM enzyme, PqqE

Wen Zhu[1,2]*, Anthony T. Iavarone[2], and Judith P. Klinman[2,3,4]*

[1]Department of Chemistry and Biochemistry, Florida State University, Tallahassee, FL 32306, United States

[2]California Institute for Quantitative Biosciences, University of California, Berkeley, CA 94720, United States

[3]Department of Chemistry, University of California, Berkeley, CA 94720, United States

[4]Department of Molecular and Cell Biology, University of California, Berkeley, CA 94720, United States

*Corresponding Authors: klinman@berkeley.edu, wzhu@chem.fsu.edu.

Table of Content

Materials and Methods

- General Information
- Protein preparation
  - PqqDAfusion construct design and protein preparation
  - PqqA purification from the PqqDAfusion protein
  - Preparation of PqqD
  - Preparation of PqqE
- SAXS sample preparation, data collection, and analysis
- PqqD and PqqE-derived peptide identification using liquid chromatography-tandem mass spectrometry
- HDX-MS sample preparation and analysis
- Structural model assembly

Supplementary Notes

Supplementary Figures

- Supplementary Figure 1
- Supplementary Figure 2
- Supplementary Figure 3
- Supplementary Figure 4
- Supplementary Figure 5
- Supplementary Figure 6
- Supplementary Figure 7

Materials and Methods

General Information. Chemicals and reagents were purchased from Fisher Scientific or Sigma-Aldrich unless stated otherwise. Modified Terrific Broth media was purchased from Research Products International. Mini-PROTEAN® TGX gels were purchased from Bio-Rad (Hercules, CA). His-tagged TEV protease was prepared and purchased from the University of California, Berkeley QB3 MacroLab. Recombinant DNA constructs were synthesized by GenScript (Piscataway, NJ). Plasmid DNA sequencing was performed by the University of California Berkeley DNA sequencing facility. Mass spectrometry (MS) measurements of proteins and peptides were performed in the University of California, Berkeley QB3/Chemistry Mass Spectrometry Facility. Anaerobic experiments were conducted in the Plas-Labs Anaerobic chamber (Lansing, MI) with the O_2 level maintained under 0.5 ppm. The O_2 level in the anaerobic chamber was monitored using a Teledyne Analytical Instruments Model 3110 Portable Oxygen Analyzer (City of Industry, CA). The anaerobic sample preparation was conducted inside Coy Anaerobic Chambers with the O_2 level maintained under 0.5 ppm in the SLAC National Accelerator Laboratory. The SEC-SAXS data were collected at Stanford Synchrotron Radiation Light Source Beamline 4-2. Prism GraphPad was used for data analysis and presentation. All the structural models were analyzed and presented using Pymol and UCSF-Chimera.

Protein preparation

PqqDAfusion construct design and protein preparation. A modified construct (pET28b-DAfus3) was designed (Supplementary Figure 1c) based on previously published methods[1]. In this new design, a TEV protease cleavage site was added between a fusion protein of *Methylorubrum extorquens* PqqD and *Methylorubrum extorquens* PqqA. NdeI and XhoI restriction sites were added at the 5'- and 3'-end of the DNA sequence and the resulting gene inserted into a pET28b vector. The plasmid was synthesized at GenScript. The DNA sequence was confirmed at the University of California Berkeley DNA sequencing facility. This plasmid was transformed into *E. coli* XL-10 competent cells for amplification and into *E. coli* BL21-Gold (DE3) competent cells for protein expression. A single colony of pET28b-DAfus3-containing *E. coli* BL21-Gold (DE3) cells was picked from a Luria Broth (LB) agar plate containing kanamycin (50 μg/mL) and cultured overnight at 37 ˚C with shaking. 10 mL of the overnight culture was diluted in 1 L of LB media containing kanamycin (50 μg/mL) and grown at 37 ˚C with 220 rpm orbital shaking until the optical density (OD_{600}) reached 0.5. The temperature of the incubator was then lowered to 18 ˚C and 100 μL of 1 M isopropyl ß-D-1-thiogalactopyranoside (IPTG) was added to induce protein expression. After 16 hours, cells were harvested by centrifugation at 3500 rpm at 4 ˚C, and the pellet was resuspended in 25 mL of lysis buffer (50 mM Tris-Cl, 150 mM NaCl, 20 mM imidazole, 2 mM TCEP, pH 7.9). The cell lysate was sonicated for 15 min on ice, and soluble protein was separated by centrifugation at 13,000 rpm for 20 min at 4 ˚C. The supernatant was loaded on a 5 mL Ni-NTA column that was pre-equilibrated with washing buffer (50 mM Tris-Cl, 150 mM NaCl, 30 mM imidazole, 2 mM 2-carboxyethyl phosphine (TCEP), pH 7.9). The column was then washed with 25 mL washing buffer, and the protein was eluted

with elution buffer (50 mM Tris-Cl, 150 mM NaCl, 300 mM imidazole 2 mM TCEP, pH 7.9). Fractions containing protein were collected and the samples containing purified protein were concentrated to ~2.5 mL using a 10 kDa Amicon Ultra Centrifugal Filter Unit (Merk Millipore, Burlington MA). The resulting solution was loaded onto a PD-10 column (Cytiva, Marlborough MA) to exchange protein into storage buffer (50 mM Tris-Cl, 150 mM NaCl, 2 mM TCEP, 10% glycerol, pH 7.9). The protein concentration was measured based on the absorbance at 280 nm using Cary UV-vis spectrometer and calculated using an extinction coefficient = 9970 $M^{-1}s^{-1}$. Approximately 60 mg of the fusion protein can be purified from a 1 L cell culture.

PqqA purification from the PqqDAfusion protein. 200 μL of PqqDAfusion protein (25 mg/mL) was incubated with 500 μL of 2 mg/mL His-tagged TEV protease at 25 ˚C for 3 hours before being cooled to 4 ˚C prior to overnight digestion. The resulting solution was mixed with denaturing buffer (8 M urea, 10 mM potassium phosphate, 10 mM beta-mercaptoethanol, pH 8.0) before being loaded onto a Ni-NTA column pre-equilibrated with denaturing buffer. The flowthrough was collected and dialyzed against lyophilization buffer (20 mM ammonium bicarbonate, 10 mM beta-mercaptoethanol pH 6.5) to remove urea. The resulting sample was lyophilized and redissolved in water containing 60 μM beta-mercaptoethanol to give a 1.1 mM PqqA stock solution, which was stored at -80 ˚C.

Preparation of PqqD. Truncated, N-terminal His-tagged *Methylorubrum extorquens* PqqD was expressed using a method similar to that used to obtain PqqDAfusion, except that the cell culture was incubated at 30 ˚C overnight after IPTG induction. Cells were harvested at 3500 rpm for 20 min at 4 ˚C, and the pellet was resuspended in 20 mL lysate buffer (50 mM Tris-Cl, 150 mM NaCl, 30 mM imidazole, pH 7.5) and then sonicated for 15 min on ice. The cell lysate was centrifuged at 13,000 rpm for 20 min, and then loaded onto a Ni-NTA column that had been equilibrated with lysate buffer. After washing with five column volumes of the lysate buffer, protein was eluted with elution buffer (50 mM Tris-Cl, 150 mM NaCl, 300 mM imidazole, pH 7.5). The fractions containing protein were collected and concentrated for buffer exchange into storage buffer (50 mM HEPES, 150 mM KCl, 5 mM TCEP, pH 7.9) using a PD-10 column. The protein concentration was determined using a Bradford assay and corrected using a conversion factor of 0.55, as reported previously.[1]

Preparation of PqqE. N-terminal Strep-tagged *Methylorubrum extorquens* PqqE was expressed and purified following previously established methods.[1] Plasmids carrying the PqqE gene (pET28-StrepPqqE) and an iron-sulfur cluster assembly gene cluster (pPH151) were co-transformed into *E. coli* BL21-Gold (DE3) competent cells to generate the PqqE expression cell line. 20 mL of cell culture was used to inoculate a 2 L of Terrific Broth containing 50 μg/mL Kanamycin and 35 μg/mL Chloramphenicol. Each 2 L of expression media was also supplied with 1 mL of 400 mM ammonium iron (III) citrate and 1 mL of 1 M magnesium sulfate. Cell cultures were incubated at 31 ˚C with agitation at 180 rpm until the OD_{600} reached 0.6. Over the next hour, the incubation temperature and rotation speed were lowered to 18 ˚C and 90 rpm, respectively, before the addition of 200

μL of 1 M IPTG and 2 mL of a freshly made, and sterilized, solution of 100 mM cysteine. The cells were pelleted after 16-18 hours by centrifugation at 3500 rpm for 20 min and stored in liquid nitrogen until purification. PqqE was purified in an anaerobic chamber at levels of < 0.5 ppm O_2. The cell pellet obtained from a 4 L expression was resuspended in 20 mL of HEPES buffer (50 mM N-2-hydroxyethylpiperazine-N'-2-ethanesulfonic acid (HEPES), 150 mM KCl, 5 mM dithiothreitol (DTT), pH 7.9) containing 10 mM ammonium iron (III) citrate, 2 mg/mL lysozyme, 5 μL Benzonase® Nuclease (MilliporeSigma, Burlington MA) and 2 mL of BugBuster® 10X Protein Extraction Reagent (MilliporeSigma). The extract containing PqqE was separated by centrifugation and loaded onto a 10 mL Strep-tactin Superflow column that had been equilibrated with HEPES buffer. After washing the column with HEPES buffer, the protein sample was eluted with HEPES buffer containing 3 mM desthiobiotin, pH 7.9, and exchanged into HEPES buffer using a PD-10 column. The protein sample was further concentrated in a 30 kDa Amicon Ultra Centrifugal Filter Unit (Merck Millipore) and stored in liquid nitrogen.

SAXS sample preparation, data collection, and analysis. PqqDE and PqqADE complexes were prepared for SAXS analysis by incubating 120 μM of PqqE, PqqD and PqqA, as needed, before being exchanged into a buffer containing 50 mM 3-[4-(2-hydroxyethyl)piperazin-1-yl]propane-1-sulfonic acid (EPPS), 300 mM KCl, 25 mM arginine, and 1 mM DTT, pH 8.0 using a 3 kDa Amicon Ultra Centrifugal Filter Unit (Merck Millipore) inside a Coy anaerobic chamber. Samples were transferred out of the chamber in sealed vials before being loaded onto a Thermo Fisher Scientific UltiMate 3000 ultra-high-performance liquid chromatography (UHPLC) system equipped with a a Superdex 200 3.2/300 size exclusion column (SEC). The column was pre-equilibrated with the degassed buffer to minimize oxidative damage to PqqE. For each complex, 50 μL of sample was injected into the column for initial separation, and the complex detected at 280 nm and 420 nm during SEC-separation in the UHPLC. SAXS data were analyzed using RAW, and electron density was calculated using DESSN.

PqqD and PqqE-derived peptide identification using liquid chromatography-tandem mass spectrometry. PqqD and PqqE-derived peptide libraries were generated using a pepsin-digestion procedure that was optimized to maximize peptide coverage across both PqqE and PqqD. Briefly, 100 μL of PqqE (290 μM), PqqD (2.27 mM), and pepsin (0.5 mg/mL) stock solutions in HEPES buffer were equilibrated in the anaerobic chamber for 30 min before mixing with H_2O buffer (50 mM HEPES, 10 mM TCEP, 30 mM KCl in H_2O, pH 7.9) to give a final concentration of 50 μM and 55 μM for PqqE and PqqD, respectively. 30 μL of citrate quenching buffer (640 mM citrate acid, 1 mM EDTA, 4 M guanidine-HCl 3 mM TCEP, pH 1) was added to the protein sample after incubation at room temperature, followed by the addition of 10 μL of 0.5 mg/mL pepsin. The digested samples were immediately moved into an ice-cold cooler and brought out of the anaerobic chamber. 80 μL of each sample was transferred into a 250-μL polypropylene vial insert (part number 5182-0549, Agilent, Santa Clara, CA) after 10 min incubation on ice outside of the anaerobic chamber before flash-freezing in liquid nitrogen. A sample containing

pepsin alone was set up as a control. These samples were analyzed using an LTQ-Orbitrap-XL mass spectrometer equipped with an electrospray ionization (ESI) source and connected in line with an UltiMate 3000 RSLCnano liquid chromatography system (LC) with a C18 column (Acclaim PepMap 100, length: 150 mm, inner diameter: 0.075 mm, particle size: 3 μm (Thermo Fisher Scientific, Waltham, MA). Peptide fragments derived from PqqD, PqqE, and pepsin were separated and analyzed as described before.[2,3] In total, a library of 123 PqqD-derived peptides and 203 PqqE-derived peptides was used as the reference for the HDX-MS analysis.

HDX-MS sample preparation and analysis. For the HDX samples containing only PqqD or PqqE, the protein stock solutions were equilibrated in the anaerobic chamber for 30 min before mixing with D_2O buffer (50 mM HEPES, 10 mM TCEP, 30 mM KCl in D_2O, pD 7.9) in a final concentration of 50 μM and 55 μM for PqqE and PqqD, respectively, in a 100 μL-labeling reaction, respectively. 30 μL of citrate quenching buffer (640 mM citric acid, 1 mM EDTA, 4 M guanidine-HCl, 3 mM TCEP, pH 1) was added to the protein sample after labeling for 2 min, 9 min, 50 min, and 180 min at room temperature in the anaerobic chamber followed by addition of 10 μL of 0.5 mg/mL pepsin. Pepsin-digested samples were moved to an ice-cold cooler immediately and brought out of the anaerobic chamber. 80 μL of each sample was transferred to the MS sample tube after 10 min incubation on ice in the atmosphere before flash-freezing in liquid nitrogen. The t = 0 min sample was prepared using the same procedure as the 2 min sample, except H_2O buffer was used instead of D_2O buffer. To prepare the binary, ternary and quaternary complexes in an anaerobic chamber, the purified PqqA, PqqD, PqqE, and SAM were equilibrated inside of the chamber for 45 min before mixing at room temperature. The 20 μL of PqqDE complex working stock containing 278 μM of PqqD and 258 μM PqqE was further incubated at room temperature for 45 min before adding to the 80 μL of D_2O buffer to initiate the deuterium labeling. This mixture contains 50 μM and 55 μM for PqqE and PqqD, respectively. For the PqqA- and/or SAM- containing ternary or quaternary complexes, 324 μM PqqA and/or 20 mM SAM was incubated with the PqqDE complex master mixture in a total volume of 20 μL at room temperature for 1 hour before adding 80 μL of D_2O buffer to initiate the deuterium labeling. This mixture contains 50 μM and 55 μM for PqqE and Pqq, respectively, and 65 μM and/or 4 mM SAM. The same quenching and digestion procedure was used for the protein complex samples. HDX-MS was determined in triplicate for samples prepared from the biological duplicate protein samples in separate expression and purification batches. The LC-MS method was adopted from previous publications.[2,3] It is not necessary to take the back exchange rate into account during the complex comparison since the back exchange in theory stays the same in the two sample sets regardless of the complex formation. The data reported for PqqE alone and PqqD alone were not corrected for back exchange. The MS data were analyzed using HDX WorkBench. A set, or library, of PqqD- and PqqE-derived peptides was selected and analyzed (see Supplementary Data). Several overlapping peptides were included to provide the best coverage of the entire protein. Manual curation was used to ensure consistency and accuracy of the automatic detection by the software. The

time dependency of the percentage deuterium uptake was exported from HDX WorkBench.[4] Volcano plots were generated by comparing the difference between two sets of percentage deuterium uptake, and statistically significant peptides were identified as P<0.01 in the unpaired Welch t-test[5] of the triplicate sample sets. We have also set a global deuterium uptake threshold (± 0.21 Da as the average of standard deviation across all the samples) to examine if any of the peptides identified based on individual significance is false positive. At this threshold, no false positive was identified.

Structure model assembly. To visualize the proposed structures of the protein complexes, we used Pymol and ChimeraX to create the representations. The assemblies were put together by superimposing the X-ray crystallography structure of PqqE (PDB: 6C8V), NMR structure of PqqD (PDB: 5SXY) and AlphaFold predicted PqqA structure (AF-Q49148-F1). The alignment of each component was manually adjusted using the crystal structure of CetB (PDB: 5WGG) as the template. These structural models serve as visual representations derived from published NMR data of PqqD[6] and the SAXS and HDX-MS results reported herein.

Supplementary Notes

Concentration of protein complex in the HDX samples. In the binary protein complex labeling experiment, 55 μM and 50 μM of PqqD and PqqE, respectively, were incubated with the D_2O-containing buffer. The calculated PqqDE complex concentration in the labeling reaction is 32-38 μM, and the free PqqD and PqqE is approximately 17-23 μM and 12-18 μM, respectively, based on the K_d determined previously (see the table below) The measurements therefore reflect a a mixture of free and bound species. An alternative protocol would have been to significantly raise the concentration of one of the protein components. This was rejected because of the requirement for large amounts of protein and also the possibility that the use of one component in large excess could skew the detection of low concentration peptides from the alternate, limiting component. In the ternary complex PqqADE, the concentration of PqqA in complex with PqqD is expected to be approximately 38 μM calculated based on the SPR measured affinities. No dissociation constant has been reported for PqqE and SAM. Based on the apparent K_M = 45 μM of SAM in another radical SAM enzyme, QueE[7], we assumed that SAM (at 4 mM) has become saturating in its ternary and quaternary complexes.

Binding affinities between PqqA, PqqD and PqqE determined by SPR and ITC previously[8]

Complex	SPR K_d (μM)	ITC K_d (μM)
PqqD + PqqE	12.5 ± 1.5	~10
PqqD + PqqA	0.39 ± 0.08	0.13 ± 0.03
PqqA/PqqD + PqqE	4.5 ± 1.5	not determined

Protein purification tags and their potential impact on the complex formation. PqqE and PqqD used in this study contain an N-terminal Strep-tag and His-tag, respectively.

The sequences of these two constructs were reported previously.[,1,8] In the HDX-analysis, the tag sequence was labeled as the negative counting from the first amino acid in the actual protein. The ITC and SPR studies on N-terminal tagged PqqD and PqqE have demonstrated that they indeed formed complexes, which is presented in the table above. In the AlphaFold predicted structures, the first 12 and 3 residues at the N-terminus of PqqE and PqqD (truncated PqqCD), respectively, are shown as loop with low confidence score, suggesting that the N-terminal is likely to be unstructured. Therefore, it is unlikely that the addition of the flexible purification tag will significantly impact the native complex formation. However, we do not exclude the possibility that the removal of the purification tag can potentially alter the value of the binding affinity, thus impacting the concentration of the protein complex in our HDX samples.

Small-angle X-ray scattering supports the proposed interaction in PqqDE and PqqADE complexes.

Although low resolution, the SAXS model is consistent with the HDX-MS measurement regarding the 'side-on' binding mode of the PqqDE complex (See Figure 2i & 3i for raw data and Supplementary Figure 4 for their analysis). The enzymes CteB and SuiB are in the same protein subfamily as PqqE and share sequence and function similarity with PqqE. However, the relative positions between the rSAM domain and the RRE domain in the crystal structure of CteB[9] and SuiB[10] are different. More specifically, the RRE domain of CteB adopts a "side-on" mode, but SuiB RRE possesses a "top-on" mode. (Supplementary Figure 4d) We calculated the theoretical SAXS trace of SuiB and CteB based on their crystal structure to further confirm our proposed "side-on" mode in the PqqDE complex using Fast SAXS Profile Computation with Debye Formula (FoXS)[11,12]. The experimental SAXS traces of PqqDE and PqqADE closely match the simulated CteB data but not SuiB (Supplementary Figure 4e) Overall, the SAXS data of PqqDE and PqqADE support a 'side-on' binding mode between PqqD and PqqE, and no large conformational changes are seen to occur when PqqA is present in the PqqADE complex.

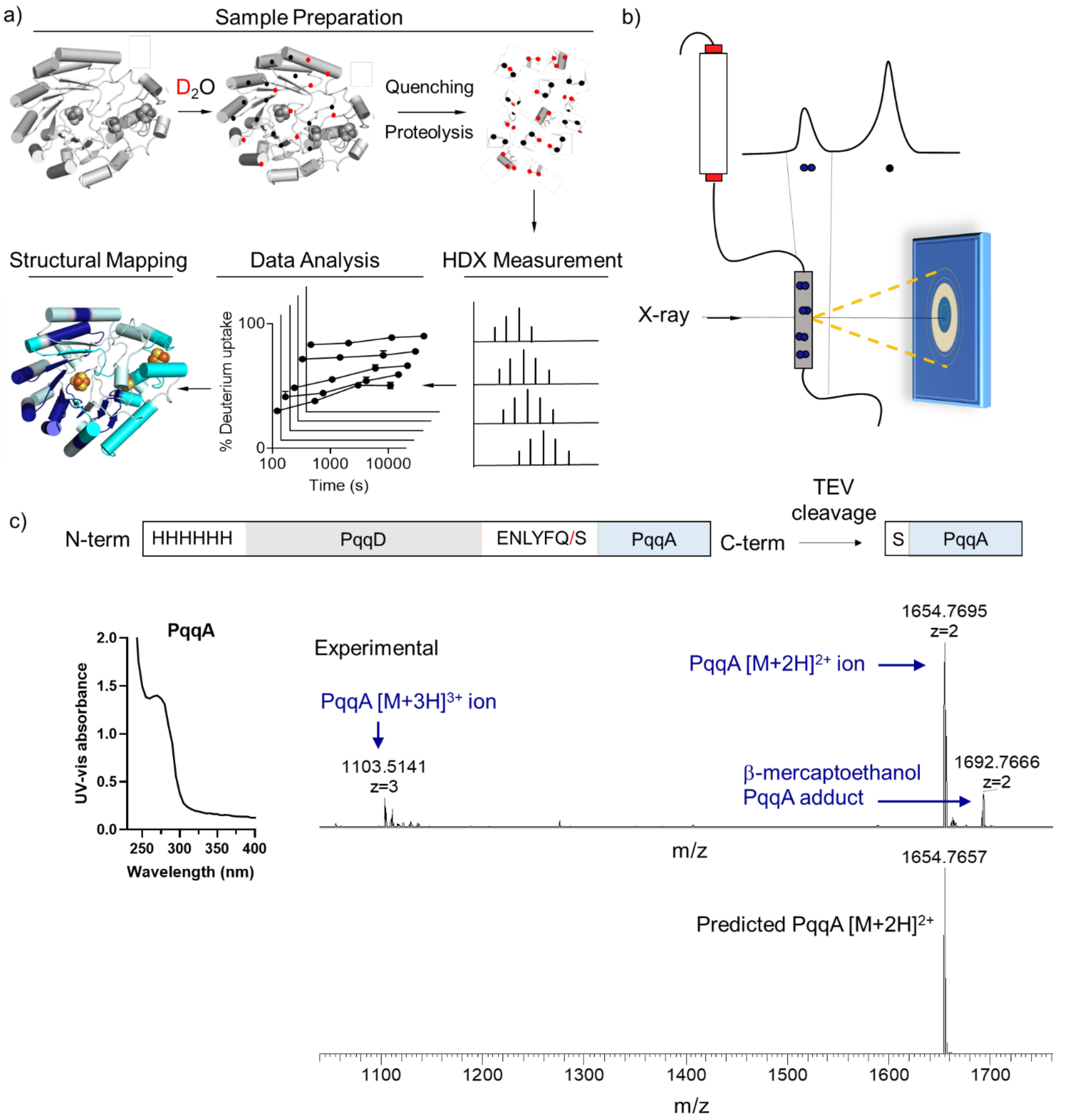

Supplementary Figure 1. HDX-MS and SEC-SAXS workflow. a) Protein samples were incubated in D_2O and quenched at various labeling times. After proteolysis, samples were analyzed by mass spectrometry. The deuterium uptake of each peptide was analyzed, classified, and mapped onto the three-demensional structures of PqqE (PDB: 6C8V) and PqqD (PDB: 5SXY). b) SEC-SAXS workflow. The protein sample was separated in ultra-high-performance liquid chromatography (UHPLC)-coupled with a size-exclusion column. Data collection started when fractions containing the protein complex flowed through the sample tubing. c) Construct for obtaining PqqA. Purified PqqA was confirmed by LC-MS.

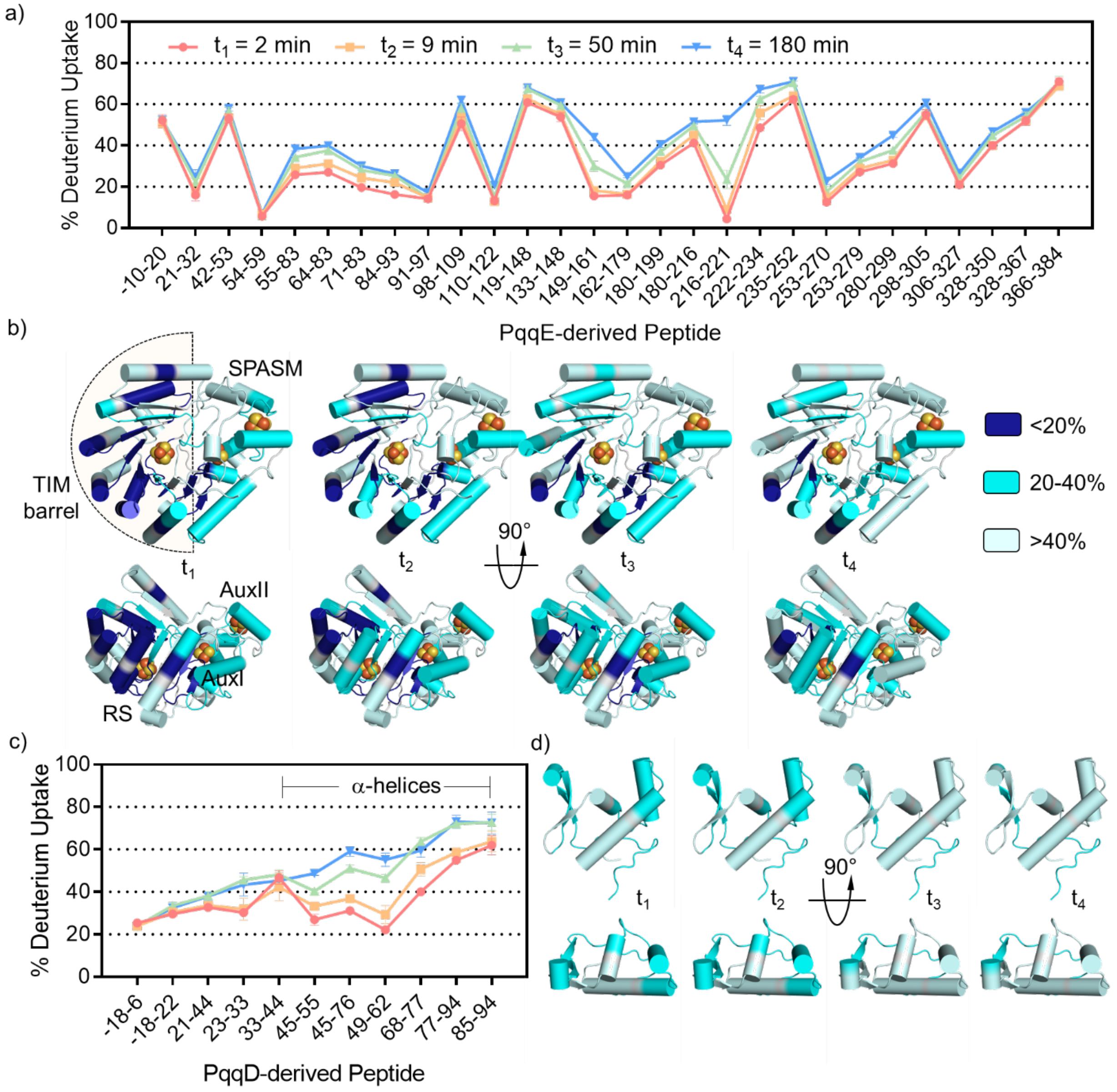

Supplementary Figure 2. HDX of the samples containing PqqE or PqqD alone. a) Percentage deuterium uptake of PqqE-derived peptides at four labeling time points. b) Mapping the HDX results onto the modeled structure of PqqE. The missing loops and the RS Fe_4S_4 cluster were modeled using AlphaFold and the CteB crystal structure. Iron-sulfur clusters are shown as spheres. c) Percentage deuterium uptake of PqqD-derived peptides at four labeling time points. d) Mapping the HDX results onto the NMR structure of PqqD. The color scheme in c) and d) is the same as a) and b).

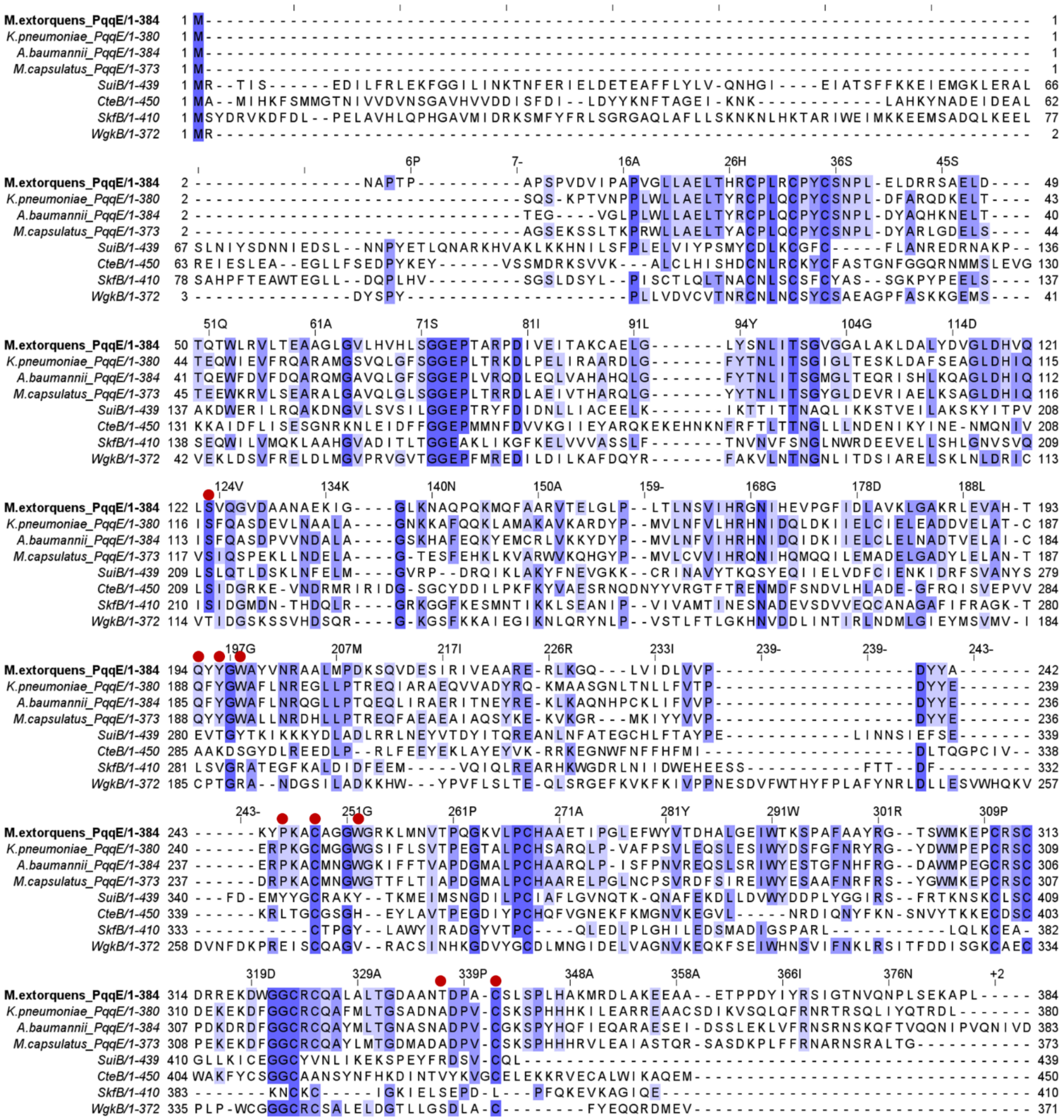

Supplementary Figure 3. Sequence alignment of PqqE and other RiPP-rSAM enzymes. Sequences of *Methylorubrum extorquens* AM1 PqqE (Uniprot P71517), Klebsiella pneumoniae PqqE (Uniprot P27507), *Acinetobacter baumannii* strain AYE PqqE (Uniprot B0V494), *Methylococcus capsulatus* Bath (Uniprot Q608P0), *Streptococcus suis* SuiB (Uniprot A0A0Z8EWX1), *Acetivibrio thermocellus* ATCC27405 CteB (Uniprot A3DDW1), *Bacillus subtilis* strain 168 SkfB (Uniprot O31423), and *Streptococcus ferus* WkgB (Uniprot A0A2X3VPX2) were aligned using MAFFT[13] and presented using Jalview[14]. Conserved residues were colored based on % identity and the red dots highlighted the residues discussed in this work.

a)

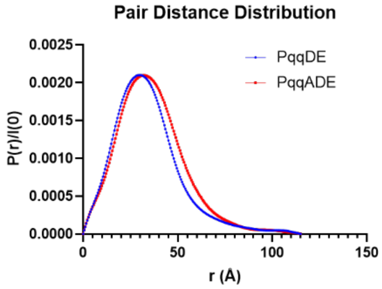

	D_{max}	R_g	I_0	Est. MW	MW
PqqADE	107.0	26.58	21.23	50216Da	59088.54
PqqDE	115.0	27.29	21.31	48474Da	55008.68

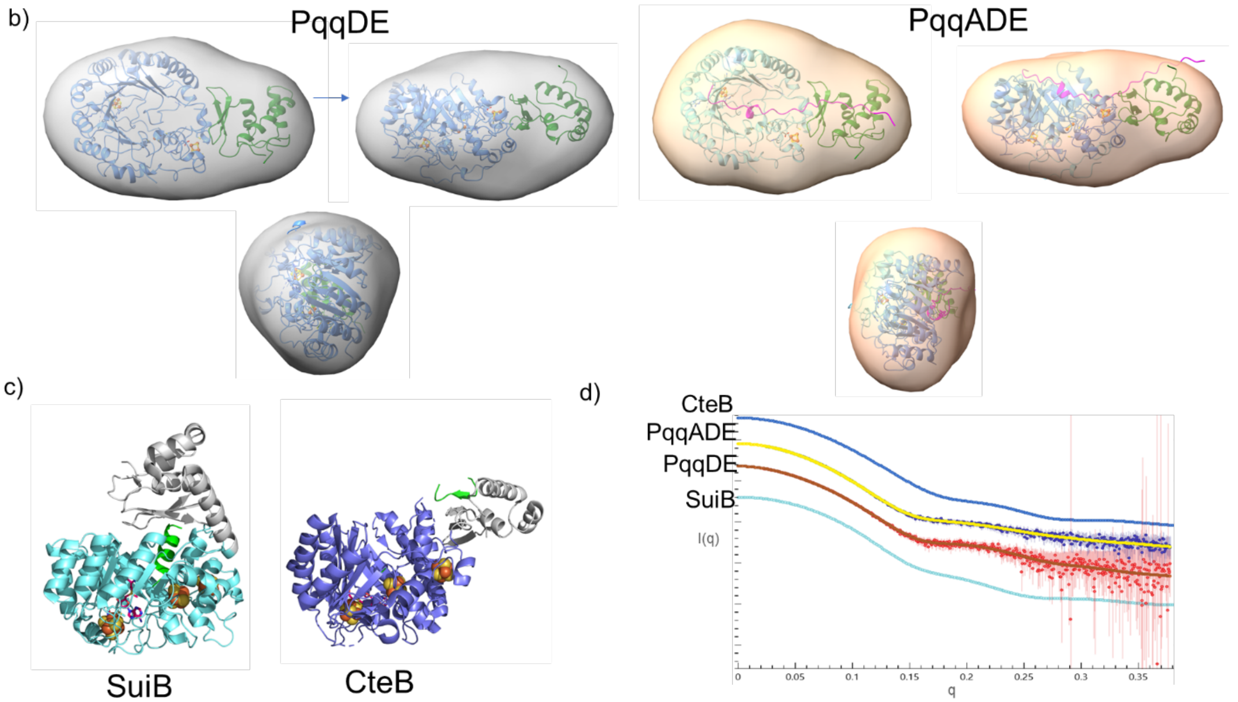

Supplementary Figure 4. Summary of SEC-SAXS data and analysis for PqqDE and PqqADE. a) Pair-distance distribution of the PqqDE and PqqADE complex. b) Electron density calculated from the SAXS data and the 'side-on' structural model. Left, gray surface represents the electron density map generated from SAXS data using DENSS[15] of PqqDE complex. The cartoon view of the 'side-on' PqqDE (PqqE in light blue and PqqD in green) is superimposed to the density map. Right, the orange surface represents the electron density map generated from the SAXS data of PqqADE, and the PqqADE structural model (PqqE in light blue, PqqA in magenta and PqqD in green) is superimposed onto the density map. c) Crystal structures of SuiB (PDB: 5VT1) and CteB (PDB: 5WGG) in complex with their substrate peptides, SuiA and CteA (green). The RRE domains in both structures are shown in gray. SuiA leader peptide interacts with the TIM barrel, and CteA leader peptide interacts with the RRE domain of CteB. d) The theoretical SAXS data calculated from the crystal structure of SuiB (cyan) and CteB (blue) in comparison to the the SAXS data of PqqDE (raw data is in red circle and fitting is in brown) and PqqADE (raw data is in dark blue circle and fitting is in yellow).

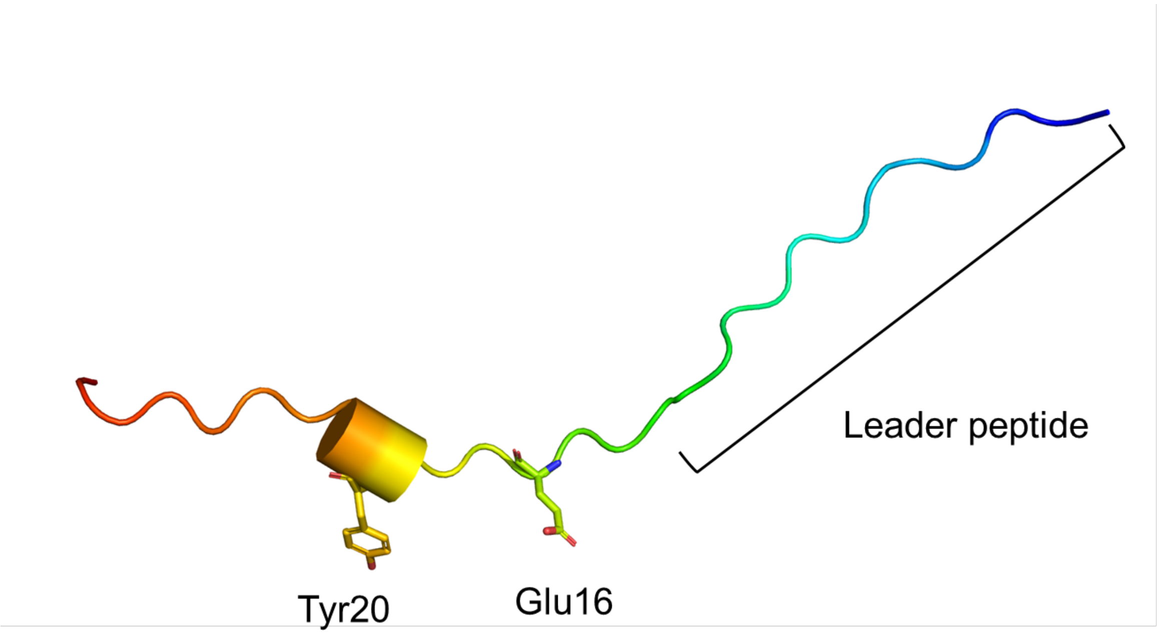

Supplementary Figure 5. AlphaFold predicted PqqA structure(AF-Q49148-F1)[16]. Glu-16 and Tyr-20 are highlighted as sticks. The structure is colored in rainbow with N-terminus being blue and C-terminus being red.

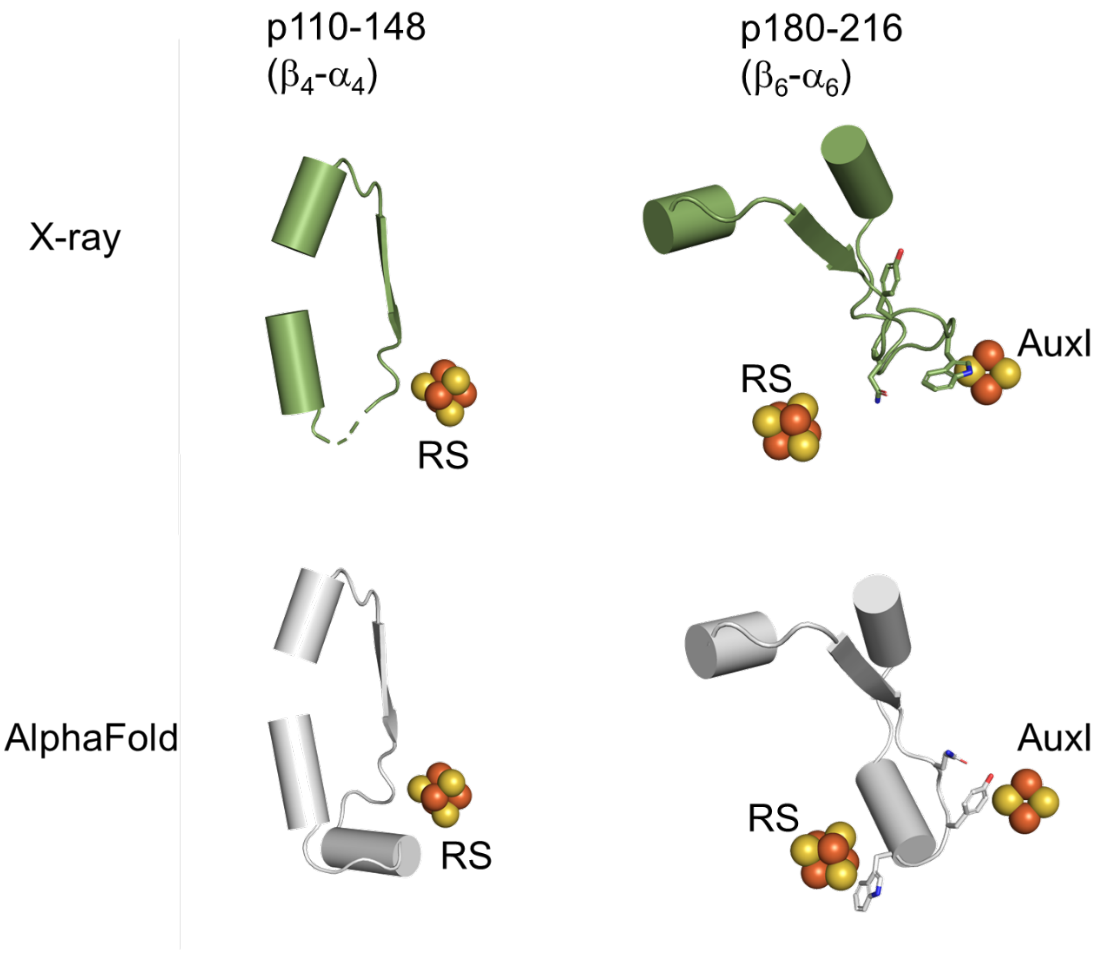

Supplementary Figure 6. AlphaFold predicted PqqE structure in comparison to the crystal structure. Residues that are not resolved in p110-148 (green) were predicted to be a helix (gray) near the RS Fe_4S_4 cluster. The QxYxW motif in [e]p180-216 is observed as a loop near the AuxI Fe_2S_2 cluster in the X-ray structure. In the AlphaFold prediction, it is a helix that relocates Trp-252 nearer the RS Fe_4S_4 cluster.

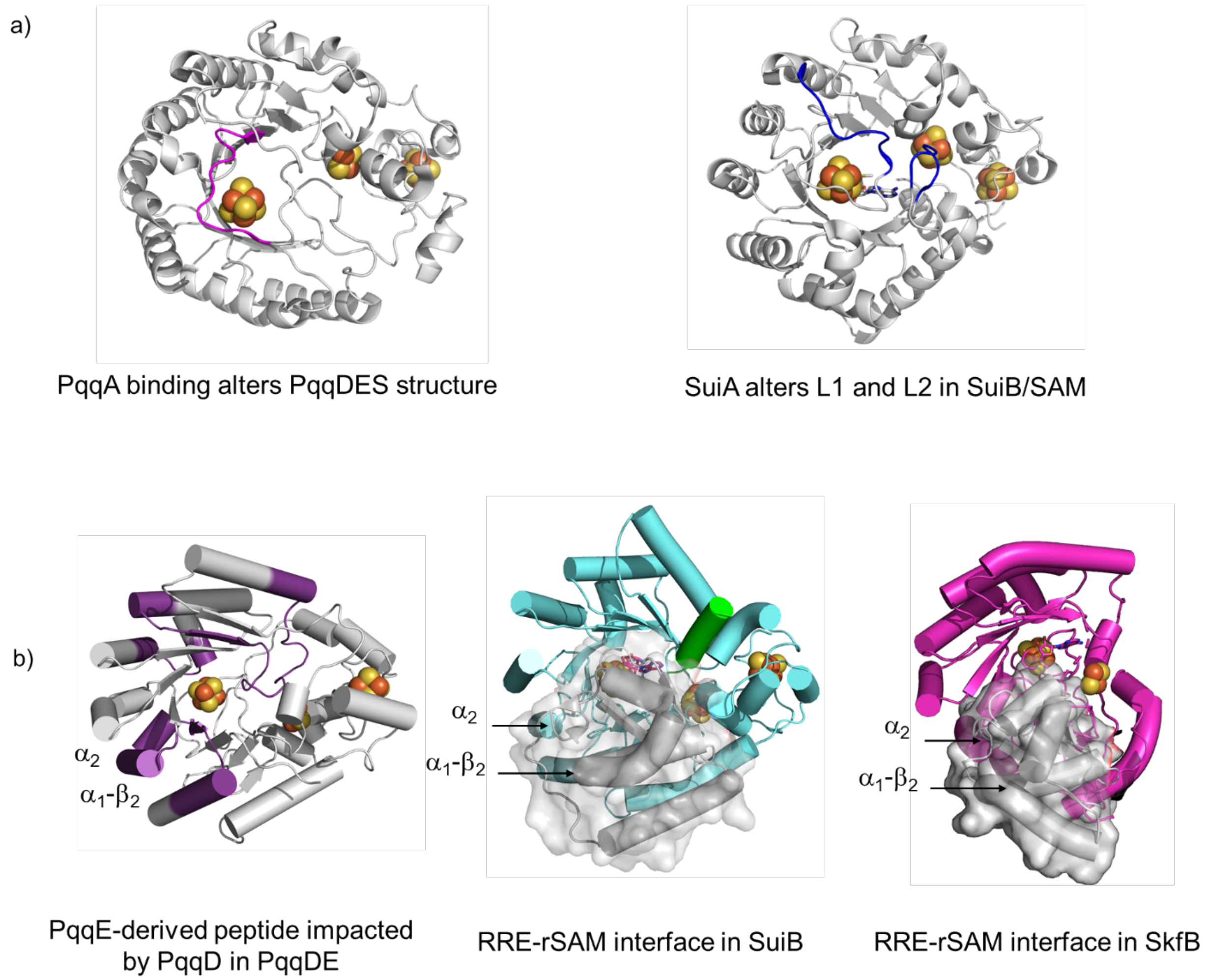

Supplementary Figure 7. Structure comparison between PqqE and SuiB. a) p21-32 (magenta) in PqqE (left) near the RS Fe_4S_4 cluster also resembles the L1 and L2 loops (blue) in SuiB (right) that are impacted by substrate binding. The iron-sulfur clusters are shown as spheres. b) The Group II peptides (purple) of PqqE in PqqDE-PqqE identified a region at α_1- β_2- α_2 that corresponds to the RRE (gray surface) binding region on rSAM (cyan) in SuiB and SkfB (magenta).

References

(1) Zhu, W.; Walker, L. M.; Tao, L.; Iavarone, A. T.; Wei, X.; Britt, R. D.; Elliott, S. J.; Klinman, J. P. Structural Properties and Catalytic Implications of the SPASM Domain Iron–Sulfur Clusters in *Methylorubrum Extorquens* PqqE. *J. Am. Chem. Soc.* **2020**, *142* (29), 12620–

(2) Thompson, E. J.; Paul, A.; Iavarone, A. T.; Klinman, J. P. Identification of Thermal Conduits That Link the Protein–Water Interface to the Active Site Loop and Catalytic Base in Enolase. *J. Am. Chem. Soc.* **2021**, *143* (2), 785–797.

(3) Gao, S.; Thompson, E. J.; Barrow, S. L.; Zhang, W.; Iavarone, A. T.; Klinman, J. P. Hydrogen–Deuterium Exchange within Adenosine Deaminase, a TIM Barrel Hydrolase, Identifies Networks for Thermal Activation of Catalysis. *J. Am. Chem. Soc.* **2020**, *142* (47), 19936–19949.

(4) Pascal, B. D.; Willis, S.; Lauer, J. L.; Landgraf, R. R.; West, G. M.; Marciano, D.; Novick, S.; Goswami, D.; Chalmers, M. J.; Griffin, P. R. HDX Workbench: Software for the Analysis of H/D Exchange MS Data. *J. Am. Soc. Mass Spectrom.* **2012**, *23* (9), 1512–1521.

(5) Welch, B. L. The Generalization of 'student's problem' hwen several different population variances are involved. *Biometrika* **1947**, *34* (1–2), 28–35.

(6) Evans, R. L.; Latham, J. A.; Xia, Y.; Klinman, J. P.; Wilmot, C. M. Nuclear Magnetic Resonance Structure and Binding Studies of PqqD, a Chaperone Required in the Biosynthesis of the Bacterial Dehydrogenase Cofactor Pyrroloquinoline Quinone. *Biochemistry* **2017**, *56* (21), 2735–2746. https://doi.org/10.1021/acs.biochem.7b00247.

(7) Lewis, J. K.; Bruender, N. A.; Bandarian, V. QueE: A Radical SAM Enzyme Involved in the Biosynthesis of 7-Deazapurine Containing Natural Products. In *Methods in Enzymology*; Elsevier, **2018**; Vol. 606, pp 95–118.

(8) Latham, J. A.; Iavarone, A. T.; Barr, I.; Juthani, P. V.; Klinman, J. P. PqqD Is a Novel Peptide Chaperone That Forms a Ternary Complex with the Radical S-Adenosylmethionine Protein PqqE in the Pyrroloquinoline Quinone Biosynthetic Pathway. *J. Biol. Chem.* **2015**, *290* (20), 12908–12918.

(9) Grove, T. L.; Himes, P. M.; Hwang, S.; Yumerefendi, H.; Bonanno, J. B.; Kuhlman, B.; Almo, S. C.; Bowers, A. A. Structural Insights into Thioether Bond Formation in the Biosynthesis of Sactipeptides. *J. Am. Chem. Soc.* **2017**, *139* (34), 11734–11744.

(10) Davis, K. M.; Schramma, K. R.; Hansen, W. A.; Bacik, J. P.; Khare, S. D.; Seyedsayamdost, M. R.; Ando, N. Structures of the Peptide-Modifying Radical SAM Enzyme SuiB Elucidate the Basis of Substrate Recognition. *Proc. Natl. Acad. Sci.* **2017**, *114* (39), 10420–10425.

(11) Schneidman-Duhovny, D.; Hammel, M.; Tainer, J. A.; Sali, A. Accurate SAXS Profile Computation and Its Assessment by Contrast Variation Experiments. *Biophys. J.* **2013**, *105* (4), 962–974.

(12) Schneidman-Duhovny, D.; Hammel, M.; Tainer, J. A.; Sali, A. FoXS, FoXSDock and MultiFoXS: Single-State and Multi-State Structural Modeling of Proteins and Their Complexes Based on SAXS Profiles. *Nucleic Acids Res.* **2016**, *44* (W1), W424–W429.

(13) Madeira, F.; Pearce, M.; Tivey, A. R. N.; Basutkar, P.; Lee, J.; Edbali, O.; Madhusoodanan, N.; Kolesnikov, A.; Lopez, R. Search and Sequence Analysis Tools Services from EMBL-EBI in 2022. *Nucleic Acids Res.* **2022**, *50* (W1), W276–W279.
(14) Waterhouse, A. M.; Procter, J. B.; Martin, D. M. A.; Clamp, M.; Barton, G. J. Jalview Version 2—a Multiple Sequence Alignment Editor and Analysis Workbench. *Bioinformatics* **2009**, *25* (9), 1189–1191.
(15) Grant, T. D. Ab Initio Electron Density Determination Directly from Solution Scattering Data. *Nat. Methods* **2018**, *15* (3), 191–193.
(16) Jumper, J.; Evans, R.; Pritzel, A.; Green, T.; Figurnov, M.; Ronneberger, O.; Tunyasuvunakool, K.; Bates, R.; Žídek, A.; Potapenko, A.; et al. Highly Accurate Protein Structure Prediction with AlphaFold. *Nature* **2021**, *596* (7873), 583–589.
