# Supplementary figures and images for: Hydrogen–Deuterium Exchange Mass Spectrometry Identifies Local and Long-Distance Interactions within the Multicomponent Radical SAM Enzyme, PqqE
Source: ACS Cent Sci. 2024 Jan 17;10(2):251–63. doi: 10.1021/acscentsci.3c01023 (PMC10906245; doi:10.1021/acscentsci.3c01023)

## Time-dependent HDX %D of PqqD-derived peptides

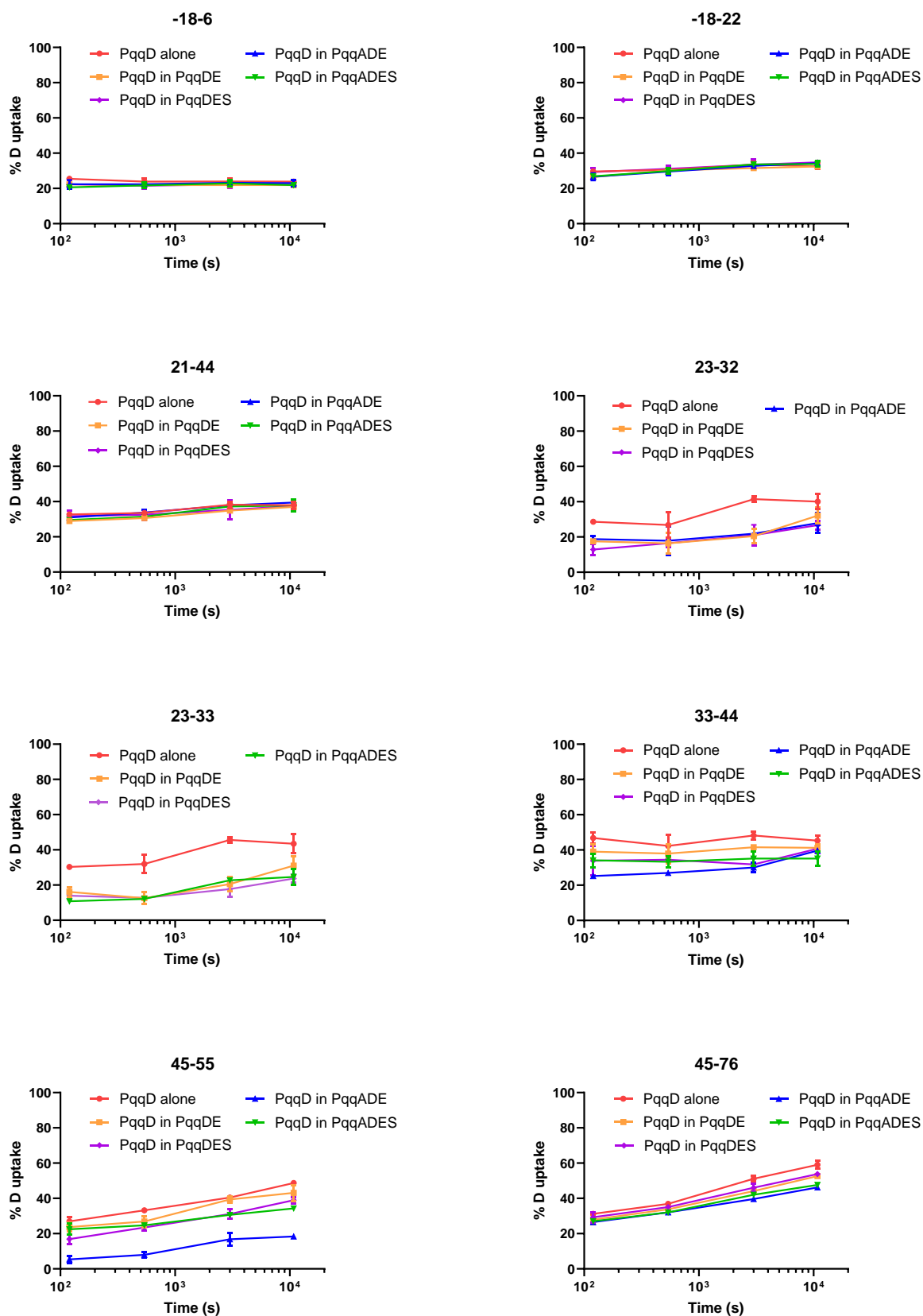

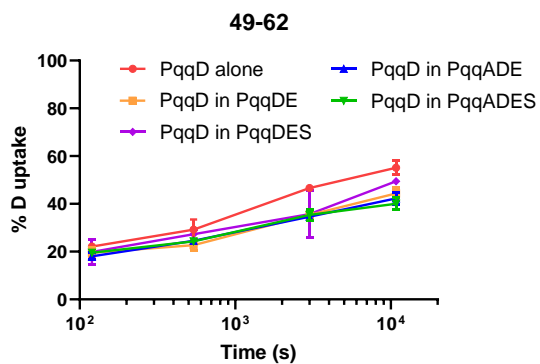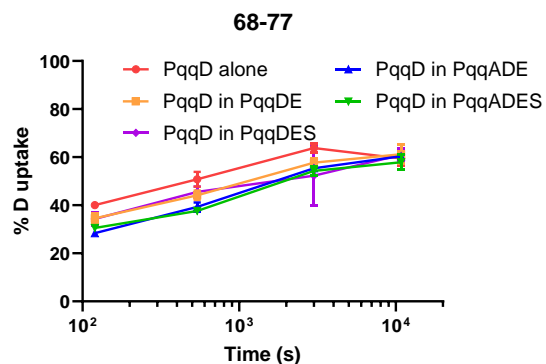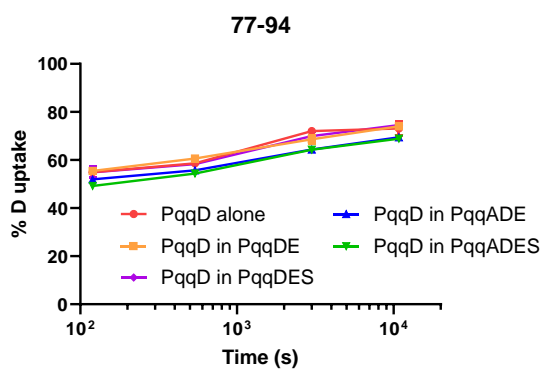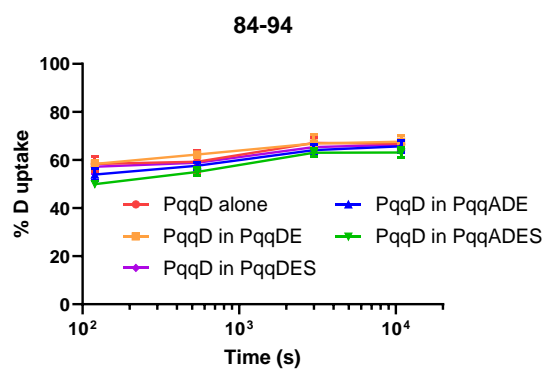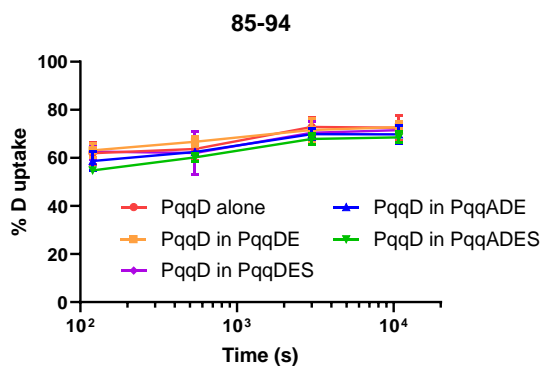

## Time-dependent HDX %D of PqqE-derived peptides

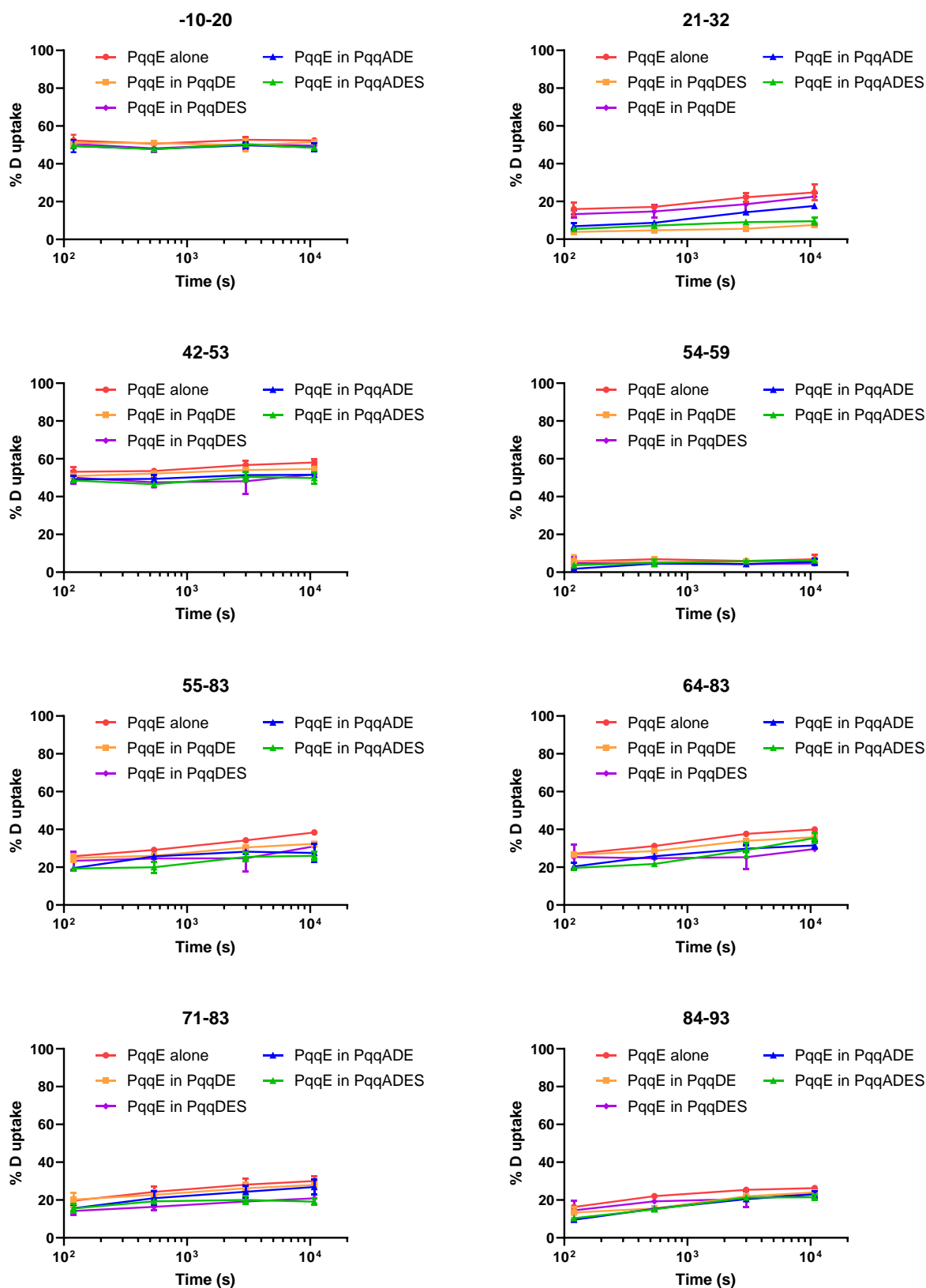

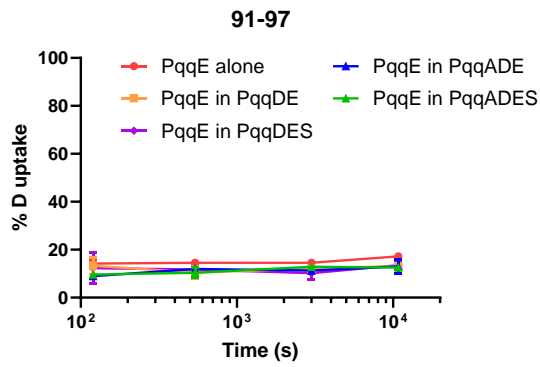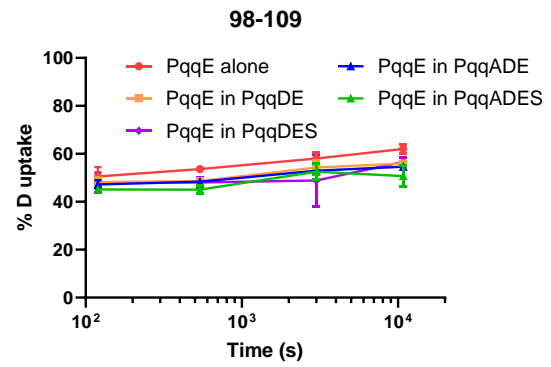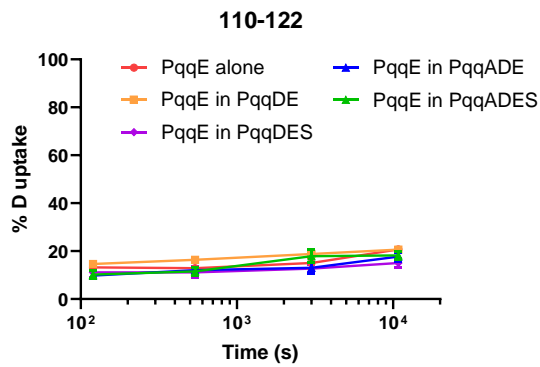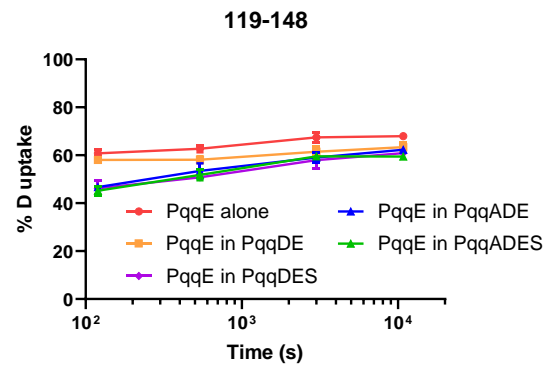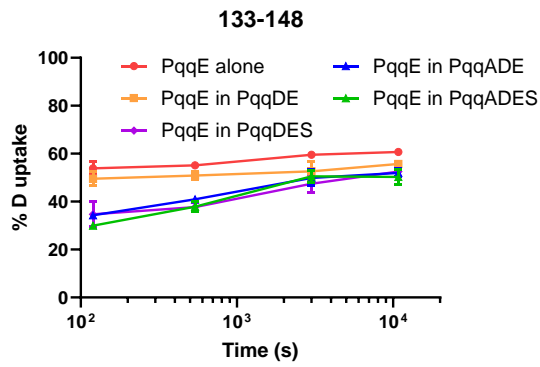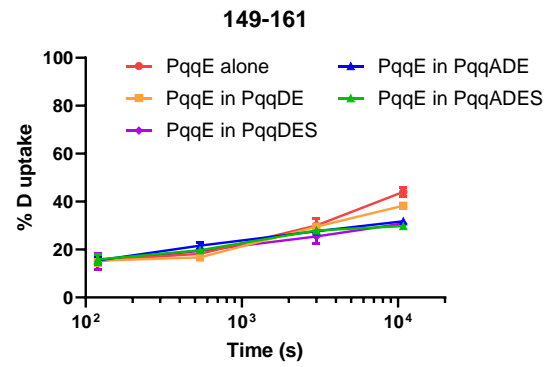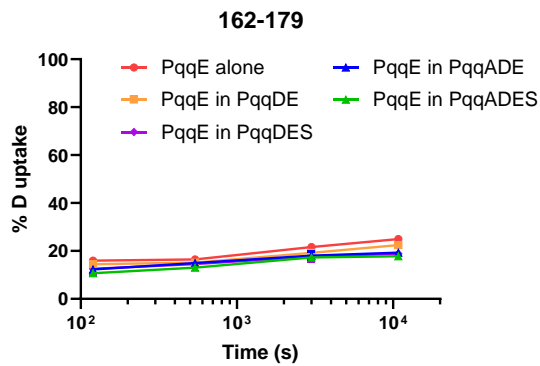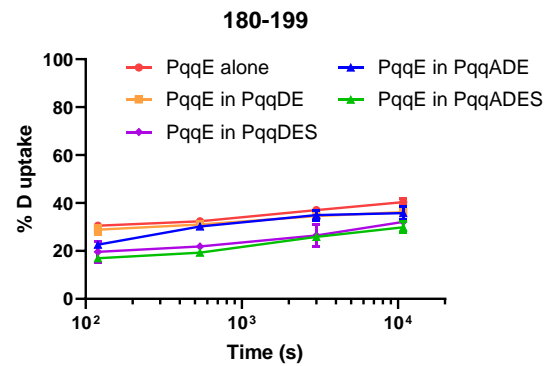

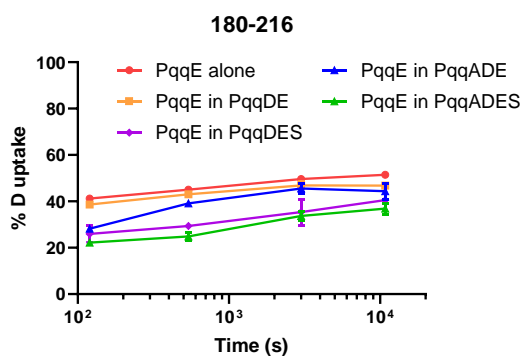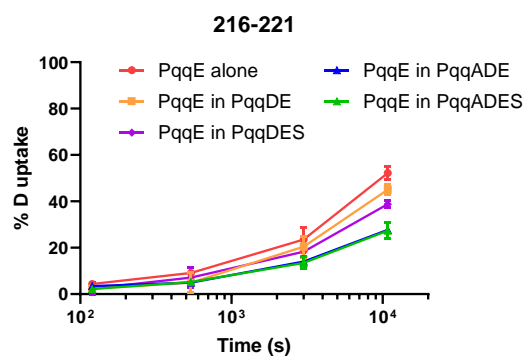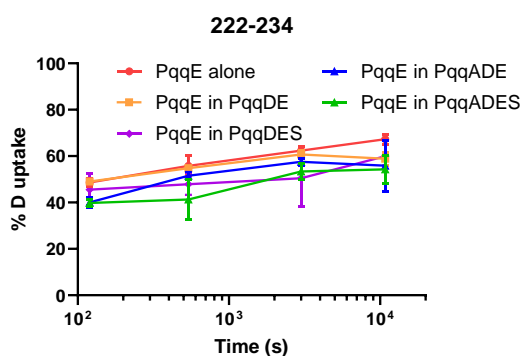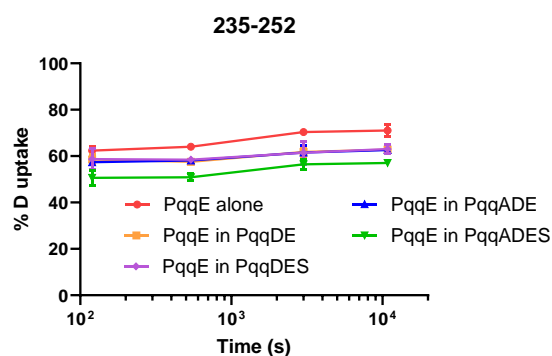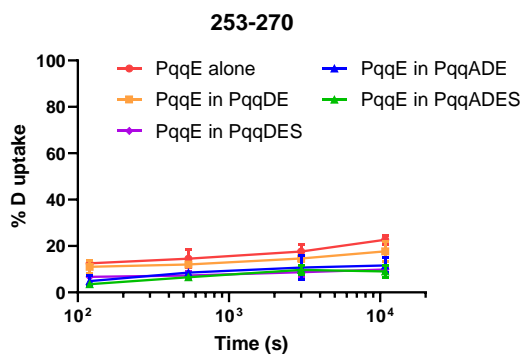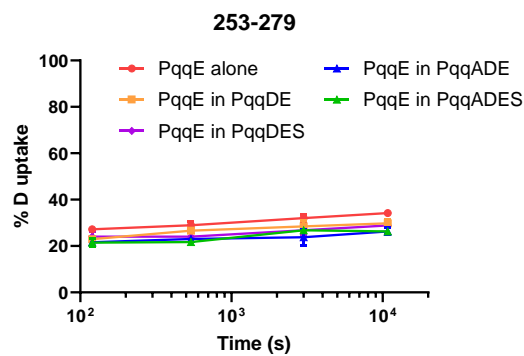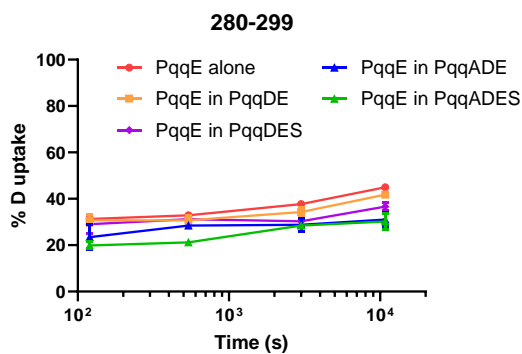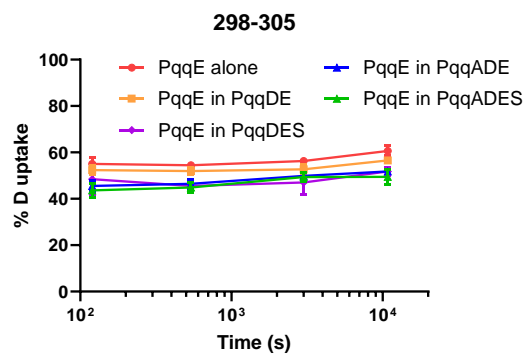

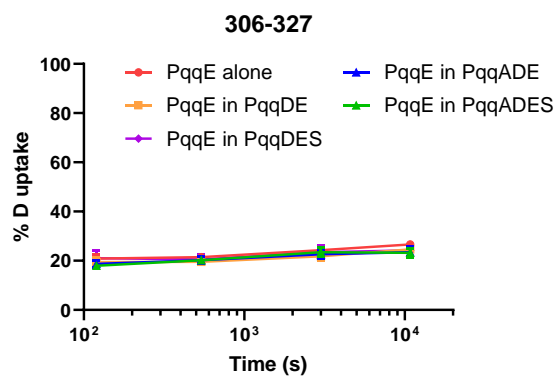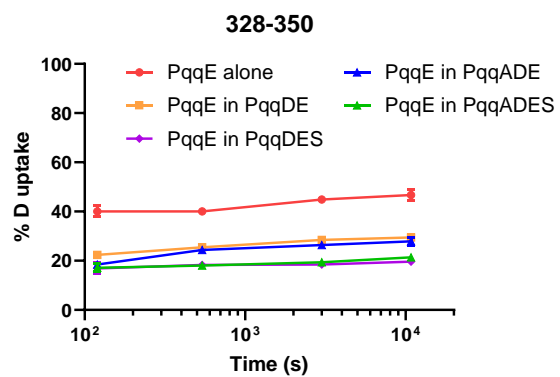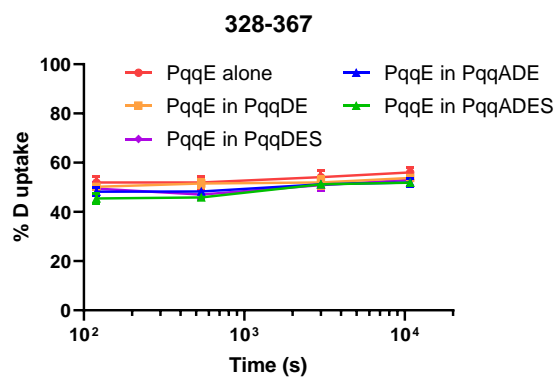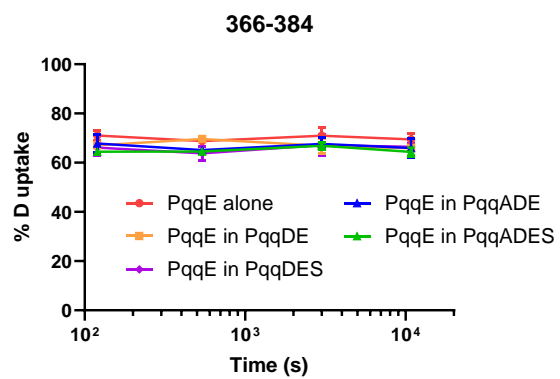

Supplement: Supplementary file 3 — oc3c01023_si_003.pdf [file oc3c01023_si_003.pdf]
